# Design, Synthesis, and Biological Evaluation of Novel Coumarin Analogs Targeted against SARS-CoV-2

**DOI:** 10.3390/molecules29061406

**Published:** 2024-03-21

**Authors:** Kirti Sharma, Manjinder Singh, Pratibha Sharma, Sumesh C. Sharma, Somdutt Mujwar, Mohit Kapoor, Krishna Kumar Mishra, Tanveer A. Wani

**Affiliations:** 1 Chitkara Institute of Engineering and Technology, Chitkara University, Rajpura 140401, Punjab, India; kirtimeetak@gmail.com (K.S.); mohit.kapoor@chitkara.edu.in (M.K.); krishna.mishra@chitkara.edu.in (K.K.M.); 2 Chitkara College of Pharmacy, Chitkara University, Rajpura 140401, Punjab, India; pratibhasharma2606@gmail.com (P.S.); somduttmujwar@gmail.com (S.M.); 3 Department of Pharmaceutical Chemistry, College of Pharmacy, King Saud University, Riyadh 11451, Saudi Arabia; twani@ksu.edu.sa

**Keywords:** SARS-CoV-2, M^Pro^, nsP3, scaffold morphing, molecular docking

## Abstract

SARS-CoV, an RNA virus, is contagious and displays a remarkable degree of adaptability, resulting in intricate disease presentations marked by frequent genetic mutations that can ultimately give rise to drug resistance. Targeting its viral replication cycle could be a potential therapeutic option to counter its viral growth in the human body leading to the severe infectious stage. The M^pro^ of SARS-CoV-2 is a promising target for therapeutic development as it is crucial for viral transcription and replication. The derivatives of β-diketone and coumarin have already been reported for their antiviral potential and, thus, are considered as a potential scaffold in the current study for the computational design of potential analogs for targeting the viral replication of SARS-CoV-2. In our study, we used novel diketone-hinged coumarin derivatives against the SARS-CoV-2 M^Pro^ to develop a broad-spectrum antiviral agent targeting SARS-CoV-2. Through an analysis of pharmacokinetics and docking studies, we identified a list of the top 10 compounds that demonstrated effectiveness in inhibiting the SARS-CoV-2 MPro virus. On the basis of the pharmacokinetics and docking analyses, the top 5 novel coumarin analogs were synthesized and characterized. The thermodynamic stability of compounds **KS82** and **KS94** was confirmed by their molecular dynamics, and the stability of the simulated system indicated their inhibitory nature. Molecules **KS82** and **KS94** were further evaluated for their anti-viral potential using Vero E6 cells followed by RT-PCR assay against SARS-CoV-2. The test compound KS82 was the most active with the potential to inhibit SARS-CoV-2 replication in Vero E6 cells. These data indicate that KS82 prevents the attack of the virus and emerges as the primary candidate with promising antiviral properties.

## 1. Introduction

RNA viruses are a group of diverse and pathogenic viruses characterized by the presence of RNA as their genetic material. Unlike DNA viruses, RNA viruses are highly prone to rapid mutation and adaptation leading to enhanced virulence with complex pathogenicity. Their inherent genetic flexibility allows RNA viruses to evolve quickly, posing ongoing challenges to public health. RNA viruses are responsible for a wide range of diseases, from the common cold to more severe illnesses like HIV, influenza, and COVID-19. Understanding the unique biology, replication mechanisms, and genomic variability of RNA viruses is crucial for the development of effective diagnostics, treatments, and vaccines [1].

COVID-19 respiratory illnesses are caused by the severe acute respiratory syndrome coronavirus, also known as SARS-CoV-2, which was first discovered in Wuhan, China, near the end of 2019. COVID-19 is having an incredible impact on human life and has turned a global public health problem into a disaster [2]. Coronaviruses belong to the Coronavirinae family. They are positive-sense RNA viruses with an envelope and a diameter of between 60 and 140 nm. SARS-CoV-2 grows in the respiratory tract, causing symptoms comparable to the common cold, such as breathing problems, nasal congestion, dry coughs, nausea, headaches, sore throat, and fever lasting many days [3]. Global efforts to quickly identify vaccines and particular antiviral medications have been sparked by the ongoing COVID-19 epidemic. SARS-CoV-2, the virus that causes COVID-19, is currently unaffected by any effective medications; nevertheless, numerous medications in diverse categories are undergoing clinical trials for therapeutic repurposing. Among the coronaviral targets that have been investigated in the past, the principal proteases (M^pro^, 3CLpro, and nsp5) have attracted a lot of focus [4,5]. RNA-dependent RNA polymerase (RdRp and nsp12), NTPase/helicase (nsp13), and papain-like protease are additional targets for coronaviral infection (PLpro, part of nsp3). Since, to our knowledge, no human host cell proteases are known to have this substrate specificity, the main protease M^pro^ is uniquely able to cleave polypeptide sequences that contain glutamine residues. Thus, the main factor causing the transmission of SARS-CoV-2 and its rapid dissemination is M^pro^ [6,7,8]. This makes M^pro^ an excellent therapeutic target. Consequently, efforts have been made to find a SARS-CoV-2 medicine that works. Numerous natural, semisynthetic, and synthetic scaffolds have been explored to inhibit the main protease, among which coumarin has been found to have effective heterocycles against this rare viral disease [9].

Coumarin is a naturally occurring substance that is formed from a variety of plant parts, as well as from several other sources like fungi and bacteria. These organic compounds exhibit an incredibly broad spectrum of biological actions with potential therapeutic benefits. As an illustration, 7-hydroxy-4-methylcoumarin was studied as a potential lead in the creation of cancer medications [10]. Different therapeutic properties of synthetic coumarins have been discovered, including antiviral, anticoagulant, anti-inflammatory, antimutagenic, anticancer, anti-tubercular, CNS stimulant, and fungicidal properties. For example, coumarin-conjugated derivatives containing benzouracil and uracil have anti-chikungunya activity. More examples are listed in the figure below, including coumarin derivatives used in the inhibition of chikv. Therefore, coumarin is receiving more interest in the field of medicinal chemistry due to its widespread distribution, the features of being a stable, easily soluble, and small-molecular-weight compound without any undesirable consequences or toxicity, and the ease of its chemical modification to develop novel semisynthetic derivatives.

**Figure 1:**
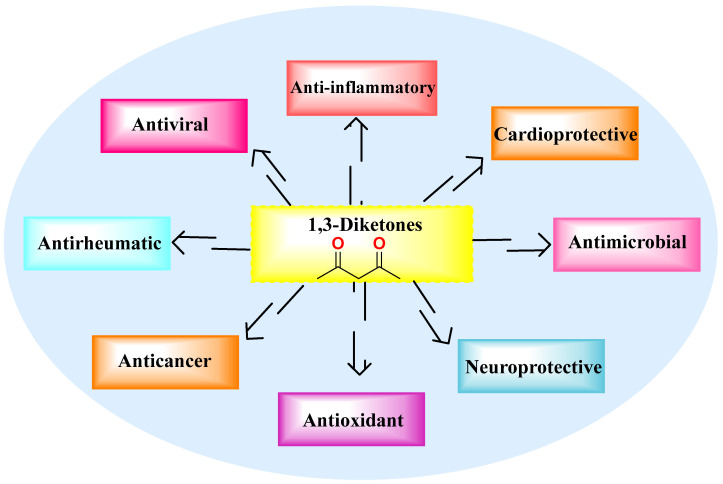
Medicinal importance of 1,3-dicarbonyls.

One of the major reasons to design coumarin derivatives containing the 1,3-dicarbonyl moiety in this study was their varied pharmacological activities, such as antioxidant, antiviral, immunomodulatory, and anti-inflammatory properties, as described in Figure 1 [11,12,13]. β-diketones are common scaffolds that can be found in many natural products and have a variety of biological functions. Numerous 1,3-diketones found in nature, such as dibenzoylmethane and curcumin, are typical examples of these substances and have been used since ancient times. Moreover, 1,3-dicarbonyls also play a very important role in the pharmaceutical industry because of their high reactivity, which helps one to design a variety of organic compounds. Various drugs containing heterocyclic moieties (pyrazole, isoxazole, carbazole, imidazole, thaizole, etc.), proven drugs against various ailments, are being synthesized via a diketone intermediate [14]. Thus, the current study aimed to design 1,3-dicarbonyl-based coumarin molecules as potential main protease inhibitors using in silico techniques for the management of the epidemic spread of SARS-CoV-2.

Coumarin-based compounds find utilization as distinctive antivirals. The current study involved the identification, screening, and designing of novel compounds with improved therapeutic profiles that were created using scaffold morphing and structure-based designing techniques [15]. Scaffold morphing, a revolutionary medicinal chemistry technique, modifies the scaffold to produce corresponding molecules with a potentially higher therapeutic profile. This approach to drug development is chemistry-driven and considers how easily novel scaffolds may be synthesized. In addition to scaffold morphing, we used the molecular docking technique to check the binding interactions between M^pro^ and 1,3-dicarbonyl-based coumarin analogs. In docking, the top 10 molecules with favorable interactions at the binding site of the SARS-CoV-2 M^pro^ were identified as potential drug candidates against this deadly virus [16]. Additionally, simulation studies for the molecules with an optimum inhibitory profile against the SARS-CoV-2 M^pro^ were also performed.

On the basis of in silico studies (scaffold morphing, ADME, and molecular docking) the top five compounds were selected and synthesized. The synthesized compounds were purified and characterized using spectral techniques [17]. The synthesized compounds were further evaluated using an in vitro SARS-CoV2 infection model in Vero E6 cells. These molecules can be used as modulators of both SARS-CoV2 and human proteins.

## 2. Results and Discussion

### 2.1. Scaffold Morphing

Using the MolOpt web server, the chemical structure of 1,3-dicarbonyl-based coumarin was determined. The seven corresponding bioisosteric sites were exchanged while conserving the dicarbonyl-coumarin system. Collectively, a library of 659 molecules was generated at the corresponding bioisosteric sites. These molecules were screened through a window of synthetic viability scores (≤3.5). With careful inspection, a total of 80 molecules were screened (Table S1). Furthermore, ADME studies were performed for the screened molecules.

### 2.2. Pharmacokinetic Predictions

The 80 screened molecules were further assessed for the determination of their drug-likeliness and ADME characteristics. The screened compounds were assessed for various physicochemical properties, like a molecular weight of 500; a QPlogP o/w of 5, 5 H-B donors, and 10 H-bond acceptors, lipophilicity, solubility on the basis of topological polar surface area (TPSA), consensus log P, ESOL LogS, etc. [18]. The screened molecules possessed an optimum profile of pharmacokinetics and drug-like properties. Overall high gastrointestinal (GI) absorption and a reasonable range of physicochemical and pharmacokinetic characteristics were displayed by these molecules (Table S2). Thus, these molecules with predicted ADME properties could be considered drug-like candidates.

### 2.3. Molecular Docking Analysis

The docking studies provided insight into the binding interactions and selectivity of the designed molecules. The molecules with significant pharmacokinetic profiles were first docked with the M^pro^ of the coronavirus [19,20].

#### Molecular Docking Analysis with M^pro^

The active site of the main protease comprised Thr24, Thr26, Phe140, Asn142, Gly143, Cys145, His163, His164, Glu166, His172, Arg188, and Gln189. The His41 and Cys145 residues form the catalytic dyad of the enzyme [21,22,23,24]. The binding energies were determined within the Cdocker module of Discovery Studio. The top molecules were selected on the basis of their binding energies (−53.57 to −45.91 Kcal mole^−1^) and as per their interactions with key residues (Table 1).

**Figure 2:**
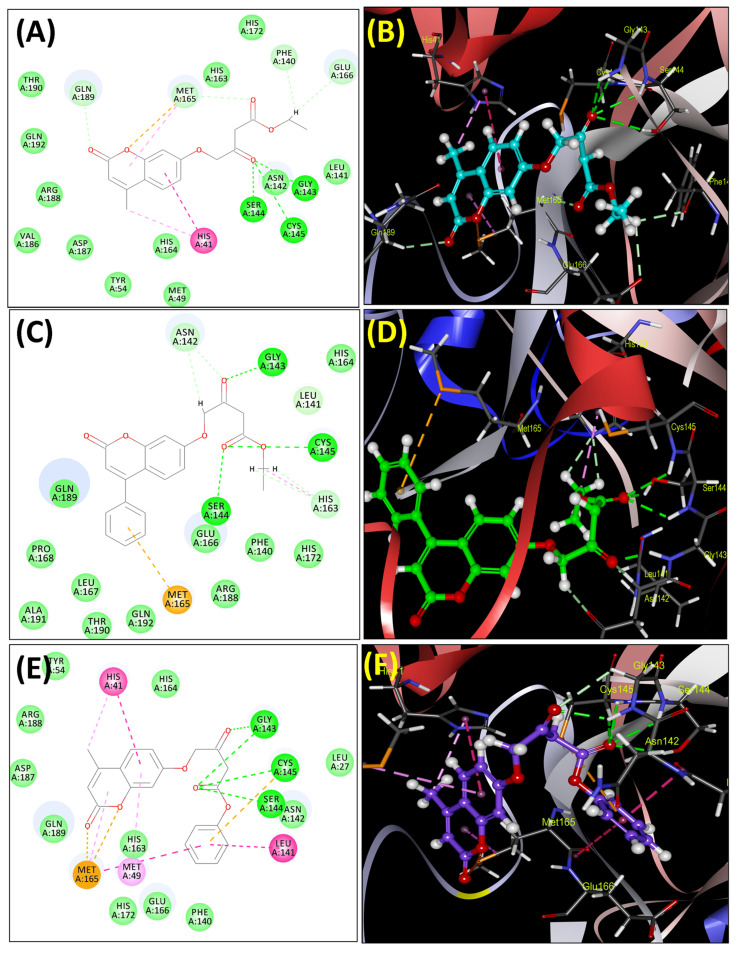
Interaction profiles of **KS81** (**A**,**B**), **KS82** (**C**,**D**), and **KS94** (**E**,**F**) with M^pro^ for the screened molecules in 2D and 3D views.

These molecules displayed interactions with the desired amino acid residues, such as Ser144, Glu166, Cys145, His41, Met49, etc. They interacted with the catalytic dyad (His41 and Cys145) via hydrogen bonding, ᴫ-ᴫ interactions, and Van der Waals forces. Most of the formulated molecules interacted with the desired amino acid residues (Ser144, Glu166, and Met49) through ᴫ-ᴫ interactions, Van der Waal interactions, alkyl interactions, or carbon–hydrogen bonding. Additional interactions with Gln189, Gly143, His163, His164, and Phe140 were also observed. Among all the molecules, **KS81** exhibited the highest binding energy of −53.57 Kcal mol^−1^ with the main protease, while **KS82** and **KS94** were next in the series with similar binding scores of −50.49 Kcal mol^−1^ and −50.02 Kcal mole^−1^, respectively. These molecules interacted with the amino acids of the catalytic dyad and additional amino acids present at the binding site (Figure 2).

All the ten leading molecules represented significant docking patterns and the corresponding critical interactions; however, two molecules, **KS82 and KS94,** demonstrated optimal docking behavior at the active site of the SARS-CoV-2 M^pro^ (Figure 2). The dicarbonyl moiety interacted with the key residues of the target protein. The aromatic rings and the lengths of the compounds are important for these interactions. Therefore, **KS82** and **KS94** were further evaluated in simulation studies.

### 2.4. Chemistry

The 1,3-dicarbonyl-fused coumarin derivatives were synthesized through the widely used method of the Pechmann condensation with slight modifications. The compounds were characterized using different spectral techniques [14,15].

#### 2.4.1. General Procedure for the Synthesis of KS(**80**–**82**)

**Scheme 1:**
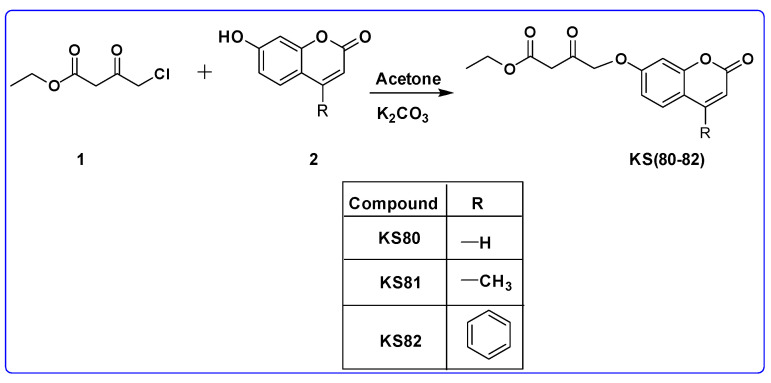
Synthetic schemes for the synthesis of 1,3-dicarbonyl-fused coumarin derivatives (**KS80–82**).

The compounds KS(**80**–**82**) were synthesized according to the general procedure given in Scheme 1, using ethyl-4-chloroacetoaceate (1) (1 mmol) and substituted 7-hydroxycoumarin (2) (1.5 mmol) stirred under reflux conditions in potassium carbonate (8 mmol) and acetone (50 mL) to afford compounds KS80–82. The crude products were treated with water, filtered, washed, dried, and recrystallized with alcohol.

#### 2.4.2. General Procedure for the Synthesis of KS92 and KS94

**Scheme 2:**
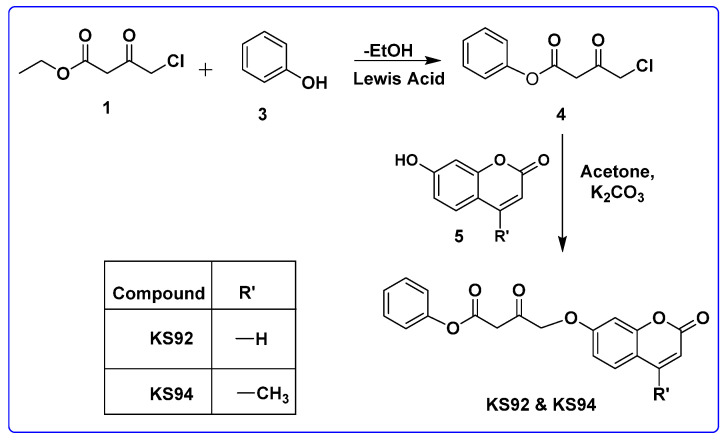
Synthetic schemes for the synthesis of 1,3-dicarbonyl-fused coumarin derivatives (**KS92** and **KS94**).

The compounds KS92 and KS94 were synthesized according to the general procedure given in Scheme 2, whereby ethyl-4-chloroacetoaceate (1) (0.1 mol) and phenol (3) (0.2 mol) were stirred in hydrochloric acid to obtain intermediate 4. Subsequently, intermediate 4 was refluxed with substituted 7-hydroxycoumarin (5) to afford the compounds KS92 and KS94. The crude products were filtered, thoroughly washed with water to remove the acid, and dried. Then, they were purified in silica columns using chloroform–methanol (9:1) as a solvent.

*Ethyl-3-oxo-4-((2-oxo-2H-chromen-7-yl)oxy)butanoate (KS80):* Light grey, solid amorphous, 78% yield, and m.p.: 192–194 °C. ^1^H NMR (500 MHz, DMSO-d_6_, δ ppm): 8.12–8.10 (1H, d, *J* = 10.9 Hz, -ArH), 7.67–7.65 (1H, d, *J* = 11.0 Hz, -ArH), 7.04 (1H, s, -ArH), 7.03–7.02 (1H, d, *J* = 4.2 Hz, -ArH), 6.17–6.15 (1H, d, *J* = 11.4 Hz, ArH), 5.18 (2H, s, -CH_2_), 4.08–4.02 (2H, q, -CH_2_), 3.55 (2H, s, -CH_2_), and 1.28–1.20 (3H, t, -CH_3_). ^13^C-NMR (125 MHz, DMSO-d_6_, δ ppm): 203.31, 168.87, 161.07, 157.87, 144.80, 129.09, 113.61, 110.50, 103.70, 82.50, 62.12, 45.02, and 15.12. IR(KBr): 3059 cm^−1^ (=C-H str, m); 1645 (C=O, s); 1605 (Ar C=C str, m); and 1128 (C-O-C). MS (ESI) *m*/*z* = 291.16 [M + H]^+^. Rf value: 0.60 (chloroform–methanol (9:1)).

*Ethyl-4-((4-methyl-2-oxo-2H-chromen-7-yl)oxy)-3-oxobutanoate (KS81):* Light yellow, solid amorphous, 70% yield, and m.p.: 209–211 °C. ^1^H NMR (500 MHz, DMSO-d_6_, δ ppm): 7.77–7.76 (1H, d, *J =* 3.9 Hz, -ArH), 7.08 (1H, s, -ArH), 7.01–7.00 (1H, d, *J* = 5.3 Hz, -ArH), 6.20 (1H, s, -ArH), 5.19 (2H, s, -CH_2_), 4.10–4.03 (2H, q, -CH_2_), 3.56 (2H, s, -CH_2_), 2.50 (3H, s, -CH_3_), and 1.22–1.20 (3H, t, -CH_3_). ^13^C-NMR (125 MHz, DMSO-d_6_, δ ppm): 204.29, 168.67, 160.97, 155.07, 152.57, 126.29, 112.91, 111.51, 103.75, 82.54, 62.10, 45.21, 20.11, and 14.91. IR(KBr): 3059 cm^−1^ (=C-H str, m); 2874 (-C-H str, m); 1651(C=O, s); 1606 (Ar C=C str, s); and 1169 (C-O-C). MS (ESI) *m*/*z* = 305.10 [M + H]^+^. Rf value: 0.62 (chloroform–methanol (9:1)).

*Ethyl-3-oxo-4-((2-oxo-4-phenyl-2H-chromen-7-yl)oxy)butanoate (KS82):* Yellowish grey, solid amorphous, 75% yield, and m.p.: 235–237 °C. ^1^H NMR (500 MHz, DMSO-d_6_, δ ppm): 7.78–7.77 (1H, d, *J* = 5.0 Hz, -ArH), 7.38–7.33 (5H, m, -ArH), 7.01 (1H, s, -ArH), 6.91–6.90 (1H, d, *J* = 6.95 *Hz*, -ArH), 6.40 (1H, s, -ArH), 5.19 (2H, s, -CH_2_), 4.00–4.01 (2H, q, -CH_2_), 3.56 (2H, s, -CH_2_), and 1.22–1.20 (3H, t, -CH_3_). ^13^C-NMR (125 MHz, DMSO-d_6_, δ ppm): 204.29, 168.69, 160.90, 155.15, 135.16, 128.21, 124.39, 112.82, 111.57, 103.79, 82.69, 62.11, 45.25, and 14.91. IR(KBr): 3080 cm^−1^ (=C-H str, m); 2954 (-C-H str, m); 1644 (C=O, s); 1602 (Ar C=C str, s); and 1178 (C-O-C). MS (ESI) *m*/*z* = 367.30 [M + H]^+^. Rf value: 0.57 (chloroform–methanol (9:1)).

*Phenyl 3-oxo-4-((2-oxo-2H-chromen-7-yl)oxy)butanoate (KS92):* Brownish yellow, solid amorphous, 75% yield, and m.p.: 246–248 °C. ^1^H NMR (500 MHz, DMSO-d_6_, δ ppm): 8.12–8.10 (1H, d, *J* = 6.05 Hz, -ArH), 7.67–7.65 (1H, d, *J* = 4.0 Hz, -ArH), 7.55–7.42 (3H, m, -ArH), 7.09 (1H, s, -ArH), 7.05–7.01 (4H, m, -ArH), 6.78–6.70 (1H, d, *J* = 6.95 Hz, -ArH), 5.19 (2H, s, -CH_2_), and 3.55 (2H, s, -CH_2_). ^13^C-NMR (125 MHz, DMSO-d_6_, δ ppm): 205.15, 167.90, 160.15, 155.11, 151.17, 144.14, 129.27, 125.35, 121.05, 113.32, 111.97, 103.25, 82.61, and 45.27. IR(KBr): 3071 cm^−1^ (=C-H str, m); 2955 (-C-H str, m); 1650 (C=O, s); 1605 (Ar C=C str, s); and 1169 (C-O-C). MS (ESI) *m*/*z* = 339.32 [M + H]^+^. Rf value: 0.48 (chloroform–methanol (9:1)).

*Phenyl 4-((4-methyl-2-oxo-2H-chromen-7-yl)oxy)-3-oxobutanoate (KS94):* Brown, solid amorphous, 85% yield, and m.p.: 251–253 °C. 1H NMR (500 MHz, DMSO-d_6_, δ ppm): 7.77–7.76 (1H, d, *J* = 4.0 Hz, -ArH), 7.56–7.43 (3H, m, -ArH), 7.25–7.24 (4H, d, *J* = 1.35 Hz, -ArH), 7.09 (1H, s, -ArH), 6.75–6.74 (1H, d, *J* = 2.0 Hz, -ArH), 6.21 (1H, s, -ArH), 5.18 (2H, s, -CH_2_), 3.59 (2H, s, -CH_2_), and 2.49 (3H, s, -CH_3_). ^13^C-NMR (125 MHz, DMSO-d_6_, δ ppm): 205.25, 167.91, 160.16, 155.19, 153.37, 151.17, 129.37, 125.31, 121.15, 112.22, 111.87, 103.21, 82.31, 45.28, and 14.82. IR(KBr): 3085 cm^−1^ (=C-H str, m); 2855 (-C-H str, m); 1641 (C=O, s); 1607 (Ar C=C str, s); and 1134 (C-O-C). MS (ESI) *m*/*z* = 353.40 [M + H]^+^. Rf value: 0.49 (chloroform–methanol (9:1)).

### 2.5. Molecular Dynamics Analysis

The MD studies were performed for the best-fit molecules (**KS82** and **KS94**) with overall significant potential interaction profiles within the viral protein. The ligand–protein interactions were inspected in dynamic motion to study the protein–ligand stability for a 100 ns time interval. The retained interactions, RMSD trajectory plot, and ligand–protein contact plot were recorded, which predicted the average change in the displacement of the protein backbone and ligand structures.

#### MD Analysis of KS82 and KS94 Complexed with M^pro^

**Figure 3:**
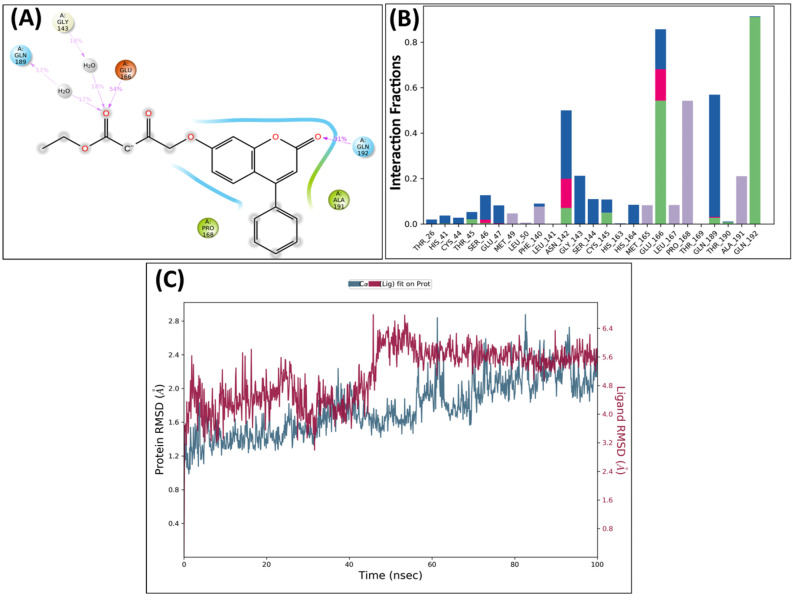
MD profile for **KS82** complexed with M^pro^ viral protein: (**A**) 2D interaction view of **KS82** with M^pro^. (**B**) Ligand–protein contact plot for **KS82** with the binding site of M^pro^. (**C**) RMSD trajectory plot for molecule **KS82**.

**Figure 4:**
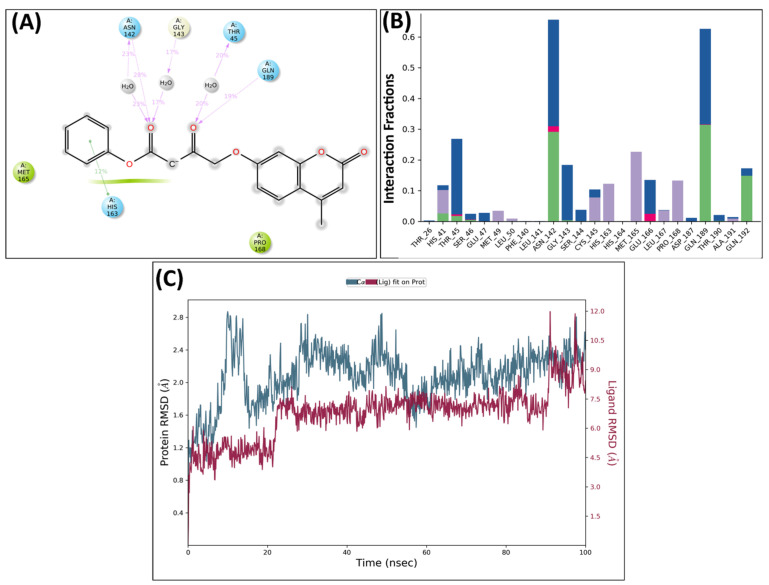
MD profile for **KS94** complexed with M^pro^ viral protein: (**A**) 2D interaction view of **KS94** with M^pro^. (**B**) Ligand–protein contact plot for **KS94** with the binding site of M^pro^. (**C**) RMSD trajectory plot for molecule **KS94**.

The stability and induced conformational adaptations upon the binding of the ligands (**KS82** and **KS94**) with the M^pro^ viral protein were analyzed with MD simulations (Figure 3 and Figure 4). **KS82 and KS94** showed interactions with the crucial amino acid residues (Glu166, Gly143, and Gln189), while **KS94** showed interactions with additional residues: His163, Met165, Asn142, and Thr45 (Figure 4A). Also, the RMSD plot (Figure 3C and Figure 4C) summarizes the stability as well as the integrity of the ligand–protein complexes during the simulations.

### 2.6. Anti-SARS-CoV2 Activity

#### 2.6.1. Cell Viability Study

The cell viability assay was performed by the widely used MTT assay in Vero E6 cells. The test compounds **KS82** and **KS94** displayed no cytotoxicity for cell lines at concentrations of up to 10 mM. The reference control remdesivir did not show any cytotoxicity to cells at 10 μM (Table 2).

#### 2.6.2. Anti-SARS-CoV2 Activity

**Figure 5:**
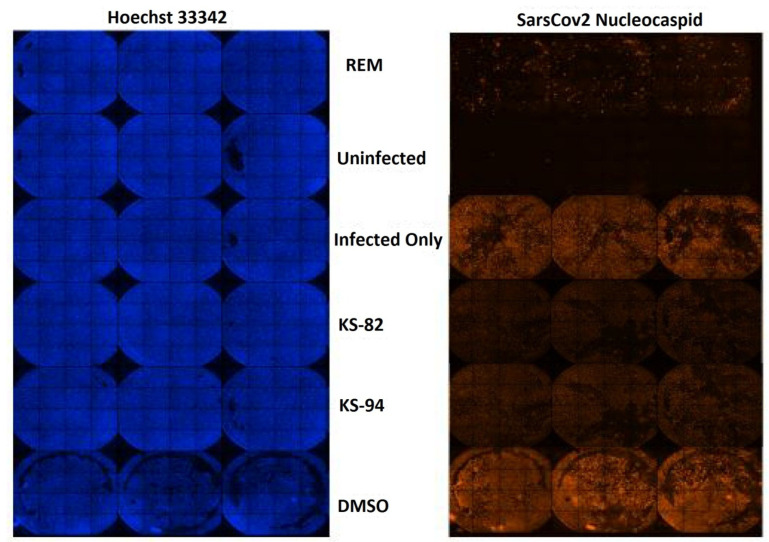
Immunofluorescence assay for validating the anti-SARS-CoV2 potency of the test compounds (**KS82** and **KS94**).

At 30 h, compound **KS82** showed a 76.23% reduction in viral RNA, and the **KS94** showed a 69.43% reduction in viral RNA. Similarly, remdesivir-treated cells showed a 96.85% reduction in viral RNA (Table 3 and Figure 5).

We observed a marked reduction (around 70–75%) in the percentage of infected cells subjected to treatment with the test drugs in comparison with the standard control. Overall, these observations suggest that the treatment with the test compounds (**KS82** and **KS94**) inhibited the viral gene expression with negligible effect on cellular viability. Thus, our in vitro results validate our in silico findings, though in vivo animal model screening is essential to establish the anti-SARS-CoV2 potential of the novel coumarin analogs. The dicarbonyl moiety and the whole coumarin aromatic ring system are important for anti-viral activity as these chemical moieties interact with the crucial amino acids of the active site.

## 3. Materials and Methods

### 3.1. Scaffold Morphing

**Figure 6:**
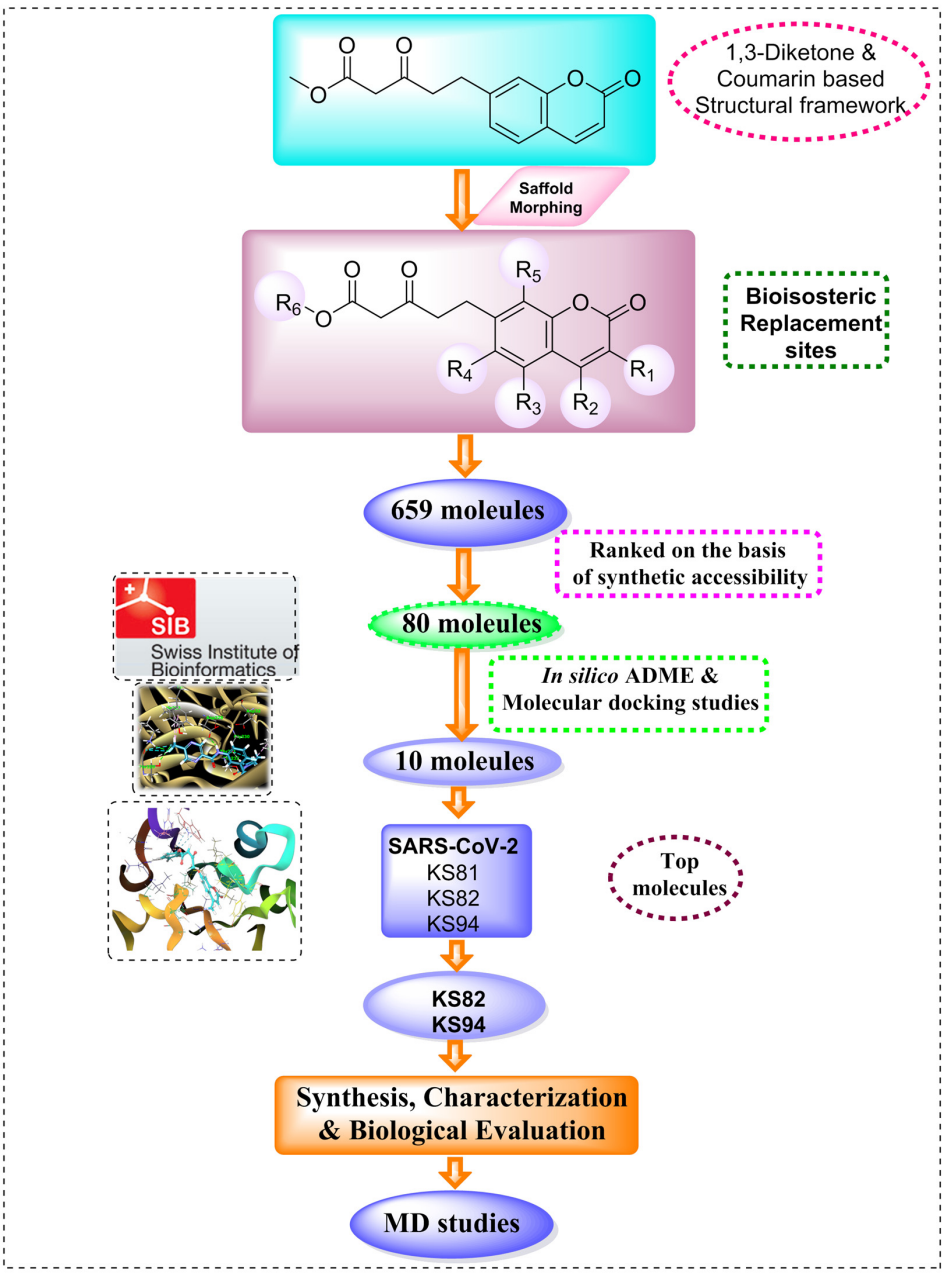
Generation of the potential antiviral molecules via various steps involved in in silico study.

Scaffold morphing, a revolutionary medicinal chemistry technique, gradually alters the original molecule to produce fresh molecules with a potentially higher therapeutic profile. The new molecules were generated as a result of the replacement of the existing groups or atoms at the varied bioisosteric sites with manual as well as scaffold morphing tools [25,26]. With a web server called MolOpt, the bioisosteric transformation of 1,3-dicarbonyls-based coumarin was carried out [27]. The seven alternate sites for the designed 1,3-diketone-coumarin scaffold were investigated. The corresponding 659 molecules decorated with the 1,3-dicarbonyl-based coumarin scaffold were generated at seven bioisosteric sites. These molecules were then sorted as a function of their “synthetic feasibility score” (≤3.5). A total of 80 molecules were obtained as per the exclusion criteria and omitting the repetitive units. Further in silico analyses were performed on these 80 molecules. The complete process embarked on during this study is outlined in Figure 6.

### 3.2. ADME Prediction

Pharmacokinetic studies ensure the anticipated pharmacological profiles of varied bioisosteres. In order to develop novel molecules, ADME profiling is the primary step in the discovery process. Moreover, early ADME estimation considerably clinically reduces pharmacokinetics-related failure. In that situation, in silico models are appropriate substitutes for experimentation [28,29]. To predict the pharmacokinetic study of the desired compounds, we used the SWISS ADME, a web server tool. It provides access to a pool of quick yet reliable predictive models for physicochemical characteristics, pharmacokinetics, and drug-likeness properties [30]. Using the swissADME program, the pharmacokinetic characteristics of the 80 screened molecules were assessed (http://www.swissadme.ch) on 11 October 2024. Their wide molecular profiles, including their physicochemical characteristics, pharmacokinetics, solubility, lipophilicity, and drug-likeness, were examined.

### 3.3. Molecular Docking

Using Biovia Discovery Studio software (v. 2019), the molecular docking studies were performed. The screened 1,3-dicarbonyl-based coumarin derivatives were docked against the main protease (M^Pro^) of the SARS-CoV-2 virus. The X-ray crystallographic structure of the M^Pro^ of SARS-CoV-2 was obtained from the Protein Data Bank (http://www.rcsb.org/pdb) accessed on 16 November 2023 with PDB ID: 6LU7 and optimized for docking analysis [31,32,33,34]. Within the ‘macromolecule module’ of the Biovia discovery studio program, the protein structure was processed [35]. The ‘small molecule module’ of the Discovery Studio program was used to prepare the ligands. Docking was performed with the ‘Dock Ligands’ protocol, and the molecular interactions of the resulting protein–ligand complexes were recorded [36,37]. The docking analysis was carried out and recorded for the best candidates. The docking results for all the docked molecules were visualized and analyzed. Finally, the two molecules with optimal pharmacokinetics and interaction behaviors against both the viral proteins were subjected to dynamic simulation studies.

### 3.4. Chemistry

All the required chemicals and solvents were purchased from various commercial distributors and were pure. With thin-layer chromatography (DC-Fertigfolien Alugram (20 × 20 cm) Kieselgel 60 F254 chromato plates), the completion of each reaction was monitored and viewed under UV light. Impurities were purified using column chromatography and recrystallization techniques with suitable solvent systems. The structures of the synthesized compounds were characterized using various spectral techniques like IR, ^1^H NMR, ^13^C NMR, and mass spectrometry.

### 3.5. Molecular Dynamics Studies

In order to explore the binding patterns of the top two molecules in a dynamic motion, simulation studies were carried out. The molecular dynamics (MD) simulation was performed using the Desmond module of Schrodinger suite 2021-1 with Linux (Ubuntu, 23.10) equipped with an NVIDIA Quadro K2200 graphics card. A simulation period of 100 ns was employed to investigate the thermodynamic stability of **KS82** and **KS94** complexed with viral proteins [38,39,40]. The ligand–protein complex was built with the explicit solvent system (TIP3P) and centered with an orthorhombic periodic boundary box. The pH adjustment was established with Na^+^ at a 0.15 M salt concentration. Using an OPLSE_2005 force field, built complex system minimization was carried out. The simulation was carried out using the NPT ensemble with a fixed temperature of 300 K using the Nosé–Hoover chain method as the thermostat, a time step of 1.0 fs, and a pressure of 1.01325 bar using a barostat (Martyn–Tobias–Klein). In order to investigate the trajectories, simulation interaction graphs were constructed. Throughout the simulation, the structural dynamic patterns of the complex were assessed as the root-mean-square deviation (RMSD) for both the components of the complexes.

### 3.6. Toxicity Testing in the Cell Culture

The cytotoxic studies of the novel coumarin analogs in Vero E6 cells (African green monkey kidney epithelial) were carried out in a 96-well plate format in 3 wells for each sample. The plates were incubated at 37 °C overnight for the formation of a monolayer. All the cells were infected using SARS-CoV-2 at an MOI of 0.01. Stocks of 10 mM of the test compounds were prepared in DMSO for further testing. The next day, the cells were incubated with the synthesized compounds. The DMSO was kept as a control. After 30 h, all cells were stained with Sytox orange and Hoechst 33,342 dyes. Pictures were taken using Image Xpress Micro confocal molecular devices at 10×, which covered 90% of the used well area, and then the plates were read spectrophotometrically. Viability was calculated against the control cells.

### 3.7. Anti-SARS-CoV2 Activity

Remdesivir was used as a positive control for SARS-CoV-2 in this assay. VeroE6 cells were kept in 96-well culture plates. After 16 h, the cells were infected using SARS-CoV-2 at a 0.1 MOI for 2 h at 37 °C. All the cells were then incubated with test molecules in a culture medium at varied non-cytotoxic concentrations. Within 5 min, the virus was added to each well at a 0.1 MOI. Control cells contained the culture medium only with a specific conc. of the vehicle. After 30 h, the cells were fixed in paraformaldehyde (4%) and 0.3% Tween-20 and stained with primary (SARS-CoV-2 nucleocapsid mouse monoclonal antibody) and secondary antibodies (anti-mouse Alexa fluor 568). Hoechst 33342 staining was used to stain the nuclei. Images were captured and analyzed at 10×, which covered 85% well area. Using the MetaXpress, micro 4 software, using a multi-wavelength cell scoring module, nucleocapsid-positive cells as well as total nuclei were counted and compared with the control [41,42].

## 4. Conclusions

Since SARS-CoV-2 poses severe health challenges and risk of death, the current study was based on designing novel coumarin-linked dicarbonylic molecules as potential antivirals against this viral entity. This work was carried out with efficient in silico tools and techniques as well as a literature analysis. A small library of 659 molecules was created, which was constricted to the 10 best molecules via various screening steps. The leading molecules showed noteworthy docking behavior at the binding site of the SARS-CoV-2 protein. The three molecules **KS81**, **KS82**, and **KS94** were identified with the highest docking scores and significant interactions with the M^pro^ of SARS-CoV-2. The optimal docking and binding potential was obtained for **KS82** and **KS94** against the SARS-CoV-2 protein. On the basis of the docking and kinetic studies, the top five novel coumarin analogs were synthesized. The molecular dynamic simulations revealed that the novel compounds **KS82** and **KS94** demonstrated significant interactions with important amino acids in the active site with a stable complex. The molecules **KS82** and **KS94** were further evaluated for their anti-viral potential using Vero E6 cells followed by RT-PCR assay against SARS-CoV-2. The test compound **KS82** was found to be the most active with the potential to inhibit SARS-CoV-2 replication in Vero E6 cells. These data indicate that **KS82** prevents the attack of the virus and emerges as the primary candidate with promising antiviral properties.

## Figures and Tables

**Table 1:** Docking scores and interactions of top 10 molecules with M^pro^.

S. No.	Molecular Structure	Cdocker Energy	Interactions Observed with the Binding Site of M^pro^
**1**	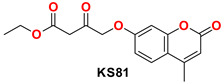	−53.57	Ser144, Gly143, **Cys145, His41**, Glu166, Leu141, Met49, Met165, and Phe140
**2**	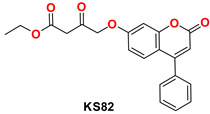	−50.49	Ser144, Pro168, Met49, Arg188, Gly143, His164, **Cys145**, and Glu166
**3**	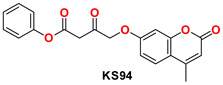	−50.02	Ser144, **Cys145***,* Gly143, Glu166, Leu141, Met49, Arg188, Glu166, Phe140, and **His41**
**4**	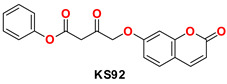	−48.53	Ser144, **Cys145**, Met49, His164, **His41**, Gly143, Glu166, Arg188, and Leu141
**5**	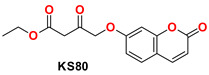	−48.16	Ser144, Gly143, **Cys145**, Tyr54, Met49, **His41**, His172, Arg188, Phe140, Leu141, and His163
**6**	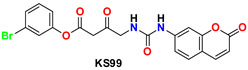	−47.70	Glu166, Ser144, **Cys145**, Gly143, Gln189, **His41**, Leu27, Pro168, and Met49
**7**	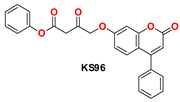	−47.14	Ser144, Gly143, **Cys145**, Met49, Met165, Met165, **His41**, Leu141, and Glu166
**8**	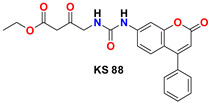	−46.85	Ser144, Gly143, **Cys145**, His164, Met49, Met49, Pro168, Leu141, **His41**, and His163
**9**	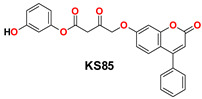	−46.31	Gly143, His164, Pro168, Met165, **Cys145**, Ser144, **His41**, Gln189, Glu166, and Met49
**10**	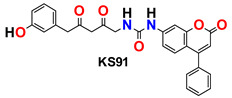	−45.91	Ser144, Gly143, Glu166, His164, Phe140, His172, Gln189, Leu141, Met49, Met165, and **His41**

**Table 2:** Cytotoxicity activity of **KS82** and **KS94**.

Compound Name	Concentration	% Cell Viability (30 h)
Remdesivir	10 µM	99.23
**KS82**	1 μL added from the stock solution into 200 μL reaction	98.34
	1 μL added from 10-times-diluted solution into 200 μL reaction	99.61
	1 μL added from 20-times-diluted solution into 200 μL reaction	107.28
**KS94**	1 μL added from the stock solution into 200 μL reaction	112.15
	1 μL added from 10-times-diluted solution into 200 μL reaction	113.57
	1 μL added from 20-times-diluted solution into 200 μL reaction	115.55

**Table 3:** % cell viability and % inhibition of **KS82** and **KS94**.

Compound Name	Concentration	% Cell Viability	% Inhibition
		30 h	
**Remdesivir**	10 µM	**99.23**	**96.85**
**KS82**	1 μL added from the stock solution	98.34	76.23
**KS94**	1 μL added from the stock solution	112.15	69.48

## Data Availability

The data presented in this study are available within the article.

## Supplementary Materials

The following supporting information can be downloaded at: https://www.mdpi.com/article/10.3390/molecules29061406/s1. Table S1. List of the screened 91 molecules on the corresponding bioisosteric sites. Table S2. The predicted Pharmacokinetic (ADME) profile of top 80 molecules.
