# Supplementary material for: Design, Synthesis, and Biological Evaluation of Novel Coumarin Analogs Targeted against SARS-CoV-2
Source: Molecules. 2024 Mar 21;29(6):1406. doi: 10.3390/molecules29061406 (PMC10976223; doi:10.3390/molecules29061406)
Supplement: Supplementary file 1 [file molecules-29-01406-s001.zip › molecules-2907905-supplementary.pdf]

Table S1. List of the screened 91 molecules on the corresponding bioisosteric sites.

S. No.	Molecule ID	Structure	S. No.	Molecule ID	Structure
1	KS1	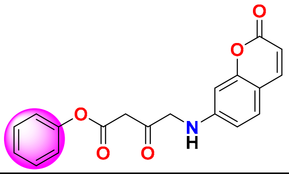	41	KS49	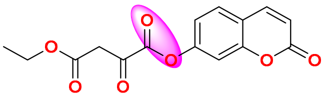
2	KS2	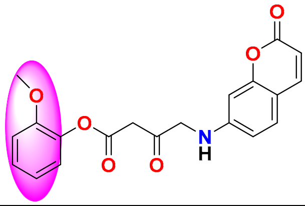	42	KS50	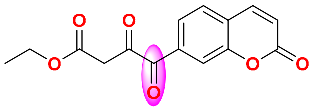
3	KS3	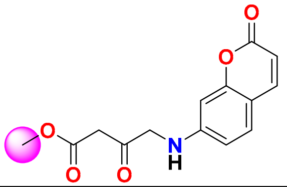	43	KS51	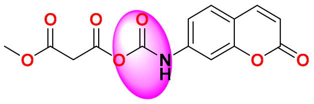
4	KS4	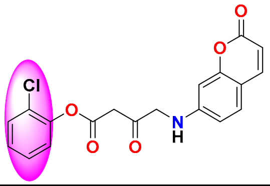	44	KS53	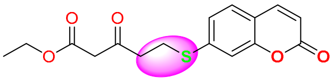
5	KS5	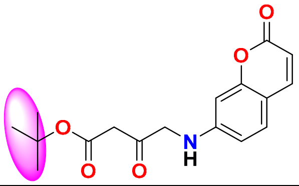	45	KS56	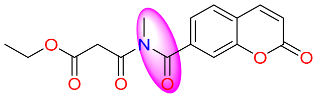
6	KS6	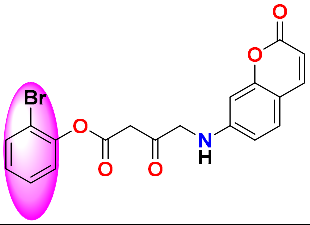	46	KS57	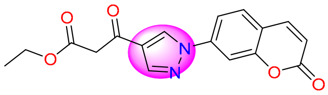
7	KS7	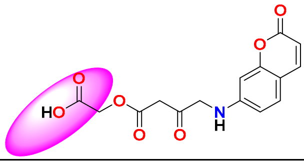	47	KS58	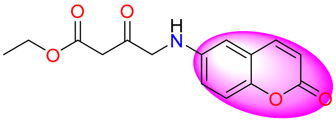
8	KS8	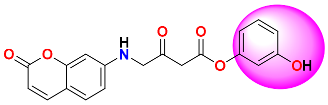	48	KS60	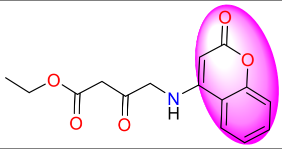

9	KS9	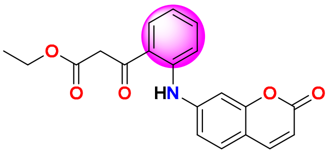	49	KS62	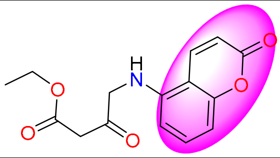
10	KS10	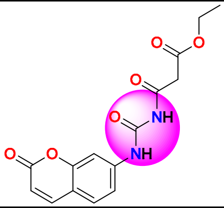	50	KS63	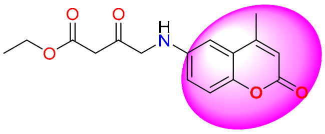
11	KS11	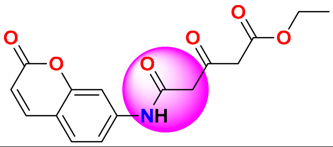	51	KS64	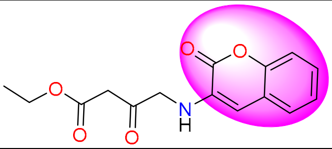
12	KS12	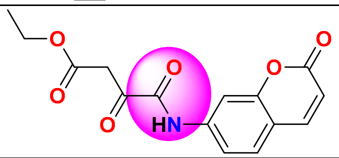	52	KS65	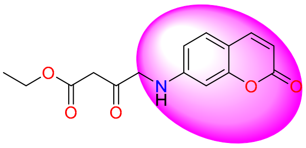
13	KS13	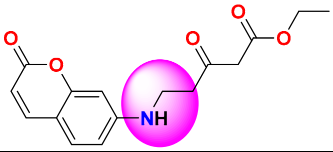	53	KS66	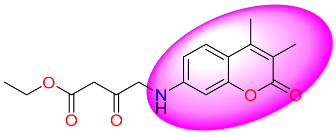
14	KS14	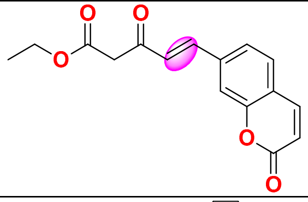	54	KS67	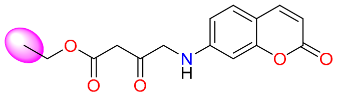
15	KS15	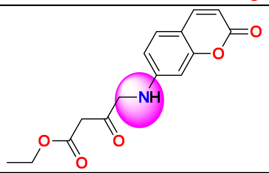	55	KS72	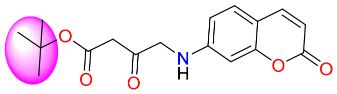
16	KS16	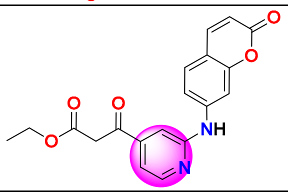	56	KS74	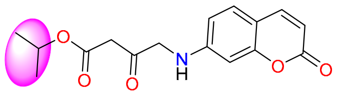
17	KS17	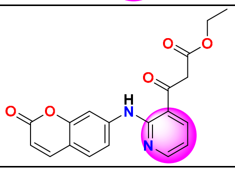	57	KS79	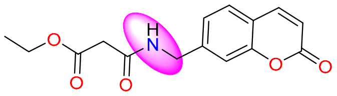
18	KS18	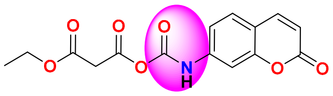	58	KS80	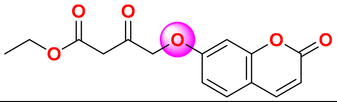

19	KS19	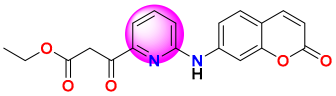	59	KS81	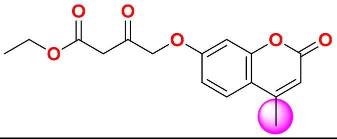
20	KS20	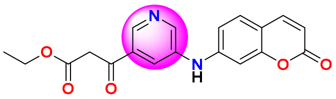	60	KS82	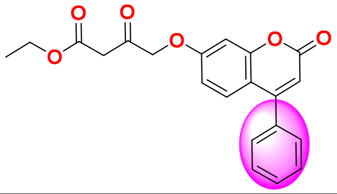
21	KS21	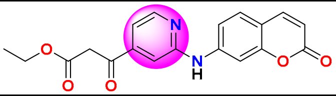	61	KS83	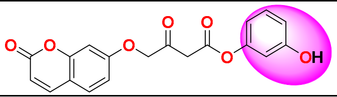
22	KS22	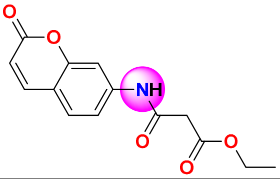	62	KS84	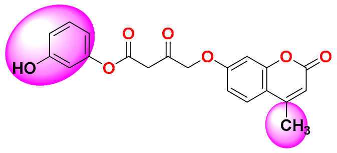
23	KS23	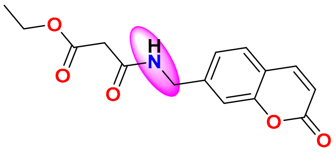	63	KS85	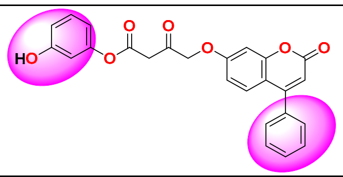
24	KS25	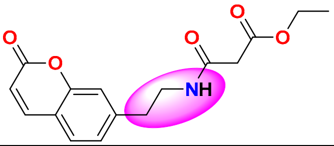	64	KS86	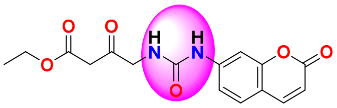
25	KS26	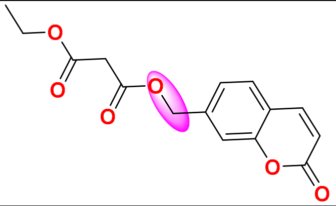	65	KS87	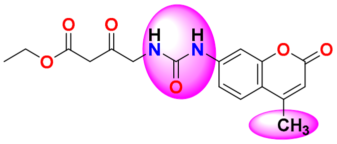
26	KS27	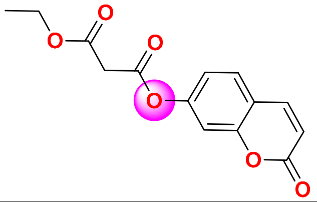	66	KS88	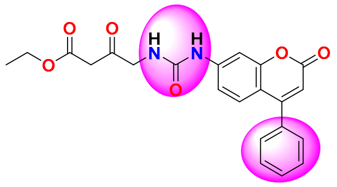
27	KS28	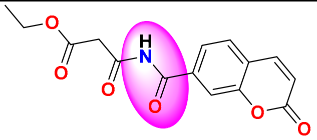	67	KS89	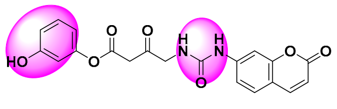

28	KS29	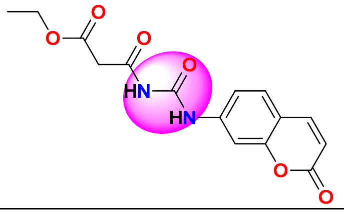	68	KS90	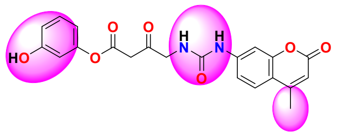
29	KS32	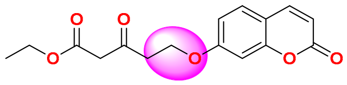	69	KS91	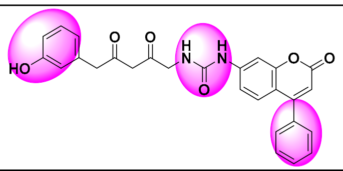
30	KS34	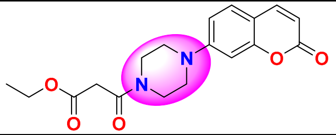	70	KS92	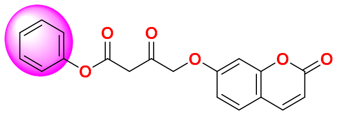
31	KS35	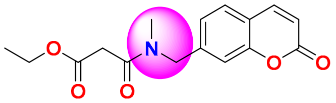	71	KS93	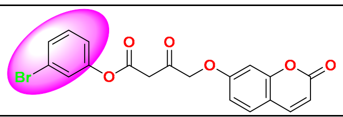
32	KS36	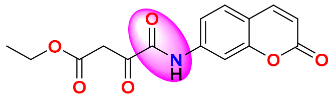	72	KS94	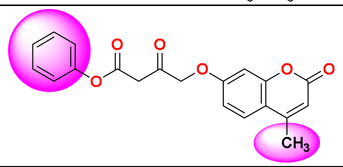
33	KS37	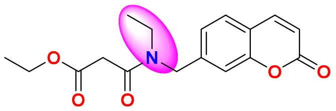	73	KS95	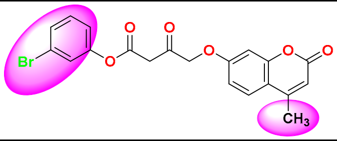
34	KS38	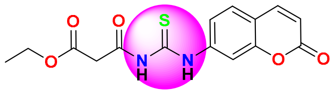	74	KS96	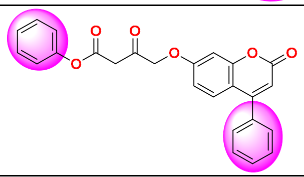
35	KS39	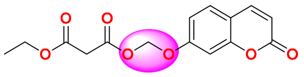	75	KS97	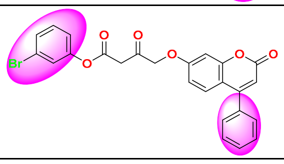
36	KS41	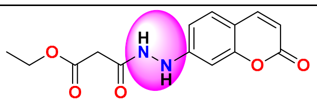	76	KS98	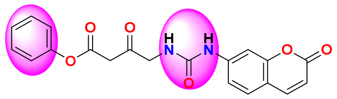
37	KS42	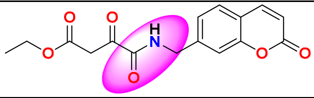	77	KS99	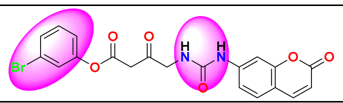
38	KS43	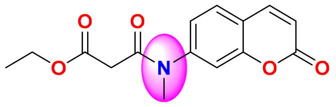	78	KS100	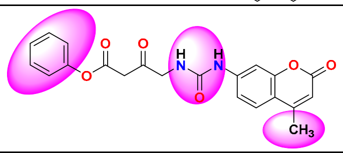

39	KS44	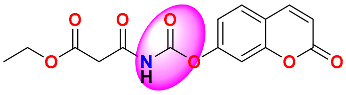	79	KS101	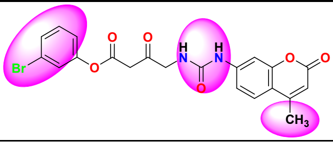
40	KS45	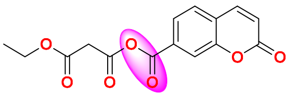	80	KS102	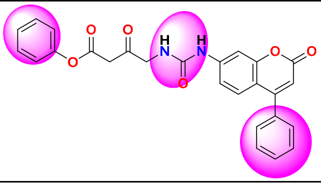
41	KS47	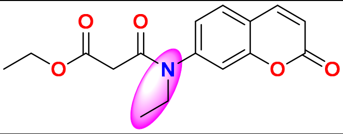			

Table S2. The predicted Pharmacokinetic (ADME) profile of top 80 molecules.

S. No.	Molecule	MW	HBA	HBD	TPSA	Consensus Log P	Silicos-IT LogSw	GI absorption	BBB permeant	Lipinski #violations
1	KS1	337.33	5	1	85.61	2.64	-6.6	High	No	0
2	KS2	367.35	6	1	94.84	2.68	-6.7	High	No	0
3	KS3	275.26	5	1	85.61	1.55	-4.5	High	No	0
4	[KS4]	371.77	5	1	85.61	3.17	-7.19	High	No	0
5	[KS5]	317.34	5	1	85.61	2.33	-5.3	High	No	0
6	[KS6]	416.22	5	1	85.61	3.31	-7.39	High	No	0
7	[KS7]	319.27	7	2	122.91	0.93	-3.87	High	No	0
8	[KS8]	353.33	6	2	105.84	2.29	-6.01	High	No	0
9	[KS9]	351.35	5	1	85.61	3.31	-6.97	High	No	0
10	[KS10]	318.28	6	2	114.71	1.32	-4.48	High	No	0
11	[KS11]	317.29	6	1	102.68	1.59	-4.83	High	No	0
12	[KS12]	303.27	6	1	102.68	1.29	-4.43	High	No	0
13	[KS13]	303.31	5	1	85.61	2.17	-5.29	High	No	0
14	[KS14]	290.27	5	2	97.64	1.45	-4.54	High	No	0
15	[KS15]	289.28	5	1	85.61	1.88	-4.9	High	No	0
16	[KS16]	352.34	6	1	98.5	2.6	-6.6	High	No	0
17	[KS17]	352.34	6	1	98.5	2.79	-6.6	High	No	0
18	[KS18]	319.27	7	1	111.91	1.73	-4.17	High	No	0
19	[KS19]	352.34	6	1	98.5	2.6	-6.6	High	No	0
20	[KS20]	352.34	6	1	98.5	2.43	-6.6	High	No	0
21	[KS21]	352.34	6	1	98.5	2.62	-6.6	High	No	0
22	[KS22]	275.26	5	1	85.61	1.74	-4.5	High	No	0
23	[KS23]	289.28	5	1	85.61	1.62	-4.9	High	No	0
24	[KS25]	303.31	5	1	85.61	1.92	-5.29	High	No	0
25	[KS26]	290.27	6	0	82.81	2.13	-4.58	High	No	0
26	[KS27]	276.24	6	0	82.81	1.98	-4.18	High	No	0
27	[KS28]	303.27	6	1	102.68	1.37	-4.43	High	No	0
28	[KS29]	318.28	6	2	114.71	1.32	-4.48	High	No	0
29	[KS32]	304.29	6	0	82.81	2.15	-4.98	High	No	0
30	[KS34]	344.36	5	0	80.06	1.65	-4.19	High	No	0
31	[KS35]	303.31	5	0	76.82	1.9	-4.56	High	No	0
32	[KS36]	303.27	6	1	102.68	1.29	-4.43	High	No	0
33	[KS37]	317.34	5	0	76.82	2.21	-4.96	High	No	0
34	[KS38]	334.35	5	2	129.73	1.93	-4.67	High	No	0
35	[KS39]	306.27	7	0	92.04	2.09	-4.32	High	No	0
36	[KS41]	290.27	5	2	97.64	1.45	-4.54	High	No	0
37	[KS42]	317.29	6	1	102.68	1.36	-4.83	High	No	0
38	[KS43]	289.28	5	0	76.82	1.86	-4.16	High	No	0
39	[KS44]	319.27	7	1	111.91	1.4	-4.17	High	No	0
40	[KS45]	304.25	7	0	99.88	1.81	-4.12	High	No	0
41	[KS49]	304.25	7	0	99.88	1.71	-4.12	High	No	0
42	[KS50]	288.25	6	0	90.65	1.46	-4.39	High	No	0

43	[KS51]	319.27	7	1	111.91	1.73	-4.17	High	No	0
44	[KS53]	320.36	5	0	98.88	2.62	-5.34	High	No	0
45	[KS56]	317.29	6	0	93.89	1.77	-4.1	High	No	0
46	[KS57]	326.3	6	0	91.4	2.02	-4.95	High	No	0
47	[KS58]	289.28	5	1	85.61	1.83	-4.9	High	No	0
48	[KS60]	303.31	5	1	85.61	2.11	-5.28	High	No	0
49	[KS62]	317.34	5	1	85.61	2.42	-5.66	High	No	0
50	[KS63]	289.28	5	1	85.61	1.88	-4.9	High	No	0
51	[KS64]	317.34	5	1	85.61	2.33	-5.3	High	No	0
52	[KS65]	303.31	5	1	85.61	2.15	-4.92	High	No	0
53	[KS66]	289.28	5	1	85.61	1.62	-4.9	High	No	0
54	[KS67]	290.27	6	0	82.81	2.13	-4.58	High	No	0
55	[KS72]	303.27	6	1	102.68	1.29	-4.43	High	No	0
56	[KS74]	290.27	5	2	97.64	1.45	-4.54	High	No	0
57	[KS79]	317.34	5	1	85.61	2.11	-5.3	High	No	0
58	[KS80]	290.27	6	0	82.81	1.98	-4.58	High	No	0
59	[KS81]	304.29	6	0	82.81	2.3	-4.97	High	No	0
60	[KS82]	366.36	6	0	82.81	3.28	-7.06	High	No	0
61	[KS83]	354.31	7	1	103.04	2.36	-5.7	High	No	0
62	[KS84]	368.34	7	1	103.04	2.7	-6.08	High	No	0
63	[KS85]	430.41	7	1	103.04	3.7	-8.15	High	No	0
64	[KS86]	332.31	6	2	114.71	1.31	-4.88	High	No	0
65	[KS87]	346.33	6	2	114.71	1.68	-5.25	High	No	0
66	[KS88]	408.4	6	2	114.71	2.52	-7.33	High	No	0
67	[KS89]	396.35	7	3	134.94	1.77	-5.98	High	No	0
68	[KS90]	410.38	7	3	134.94	2.03	-6.35	High	No	0
69	[KS91]	472.45	7	3	134.94	3.02	-8.42	Low	No	0
70	[KS92]	338.31	6	0	82.81	2.76	-6.28	High	No	0
71	[KS93]	352.34	6	0	82.81	3.12	-6.66	High	No	0
72	[KS94]	414.41	6	0	82.81	4.06	-8.74	High	No	0
73	[KS95]	417.21	6	0	82.81	3.36	-7.08	High	No	0
74	[KS96]	431.23	6	0	82.81	3.66	-7.45	High	No	0
75	[KS97]	493.3	6	0	82.81	4.63	-9.51	High	No	0
76	[KS98]	380.35	6	2	114.71	2.11	-6.57	High	No	0
77	[KS99]	394.38	6	2	114.71	2.35	-6.94	High	No	0
78	[KS100]	456.45	6	2	114.71	3.36	-9.01	High	No	0
79	[KS101]	459.25	6	2	114.71	2.73	-7.35	High	No	0
80	[KS102]	473.27	6	2	114.71	2.99	-7.72	High	No	0

Characterization of Synthesized Compounds

All the synthesized chromen derivatives were characterized by their physical properties as well as spectral analysis using IR, NMR and Mass spectroscopy.

Physicochemical properties and TLC data of synthesized compounds

Compound Codes	Molecular Formula	Molecular Weight	% Yield	m.p. (°C)	R_f value*
**KS-80**	$C_{15}H_{14}O_6$	290.27	78	192-194	0.60[a]
**KS-81**	$C_{16}H_{16}O_6$	304.30	70	209-211	0.62
**KS-82**	$C_{21}H_{18}O_6$	366.37	75	235-237	0.57
**KS-92**	$C_{19}H_{14}O_6$	338.32	70	245-248	0.48
**KS-94**	$C_{20}H_{16}O_6$	352.34	85	251-253	0.49

*Solvent System: Chloroform:Methanol (9:1)

General procedure for the synthesis of KS(**80-82**)

The compounds KS(**80-82**) was synthesized according to the general procedure given in scheme 1, using ethyl-4-chloroacetoaceate (1) (1 mmol) and substituted 7-hydroxycoumarin (2) (1.5 mmol) was stirred under reflux conditions in potassium carbonate (8 mmol) and acetone (50 ml) to afford compounds KS80-82. The crude products were treated with water, filtered, washed, dried and recrystallized with alcohol.

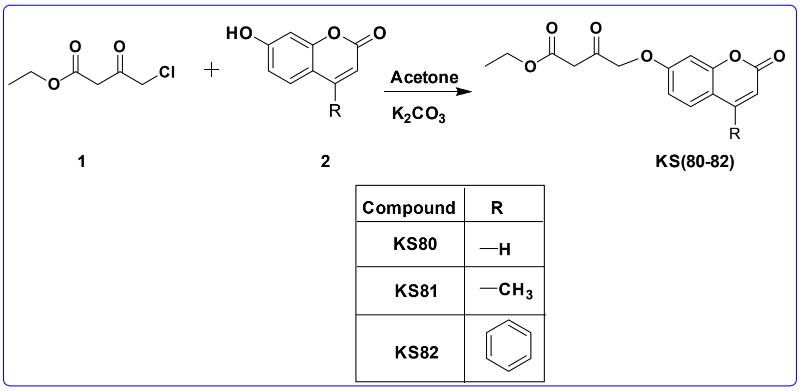

2.4.2. General procedure for the synthesis of KS92 and KS94

The compound KS-92 and KS-94 were synthesized according to the general procedure given in scheme 2, ethyl-4-chloroacetoaceate (1) (0.1 mol) and phenol (3) (0.2 mol) was stirred in hydrochloric acid to get the intermediate 4. Subsequently, the intermediate 4 is refluxed with substituted 7-hydroxycoumarin (5) to afford the compound KS-92 and KS-94. The crude products were filtered, thoroughly washed with water to make it free from acid and dried. Then, purified on silica columns using $CHCl_3$: MeOH (9:1) as solvent.

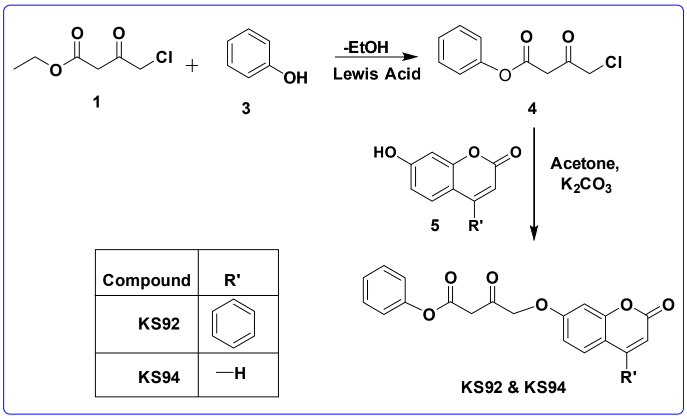

Mechanism I

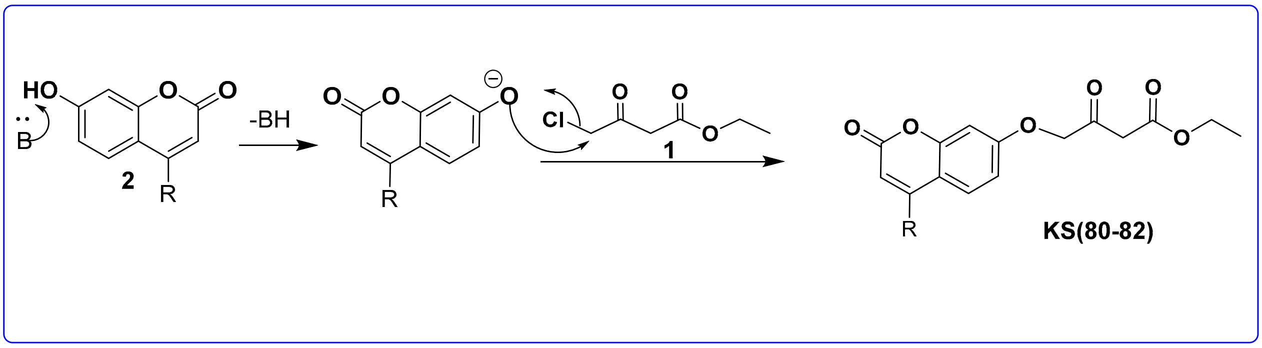

Mechanism II

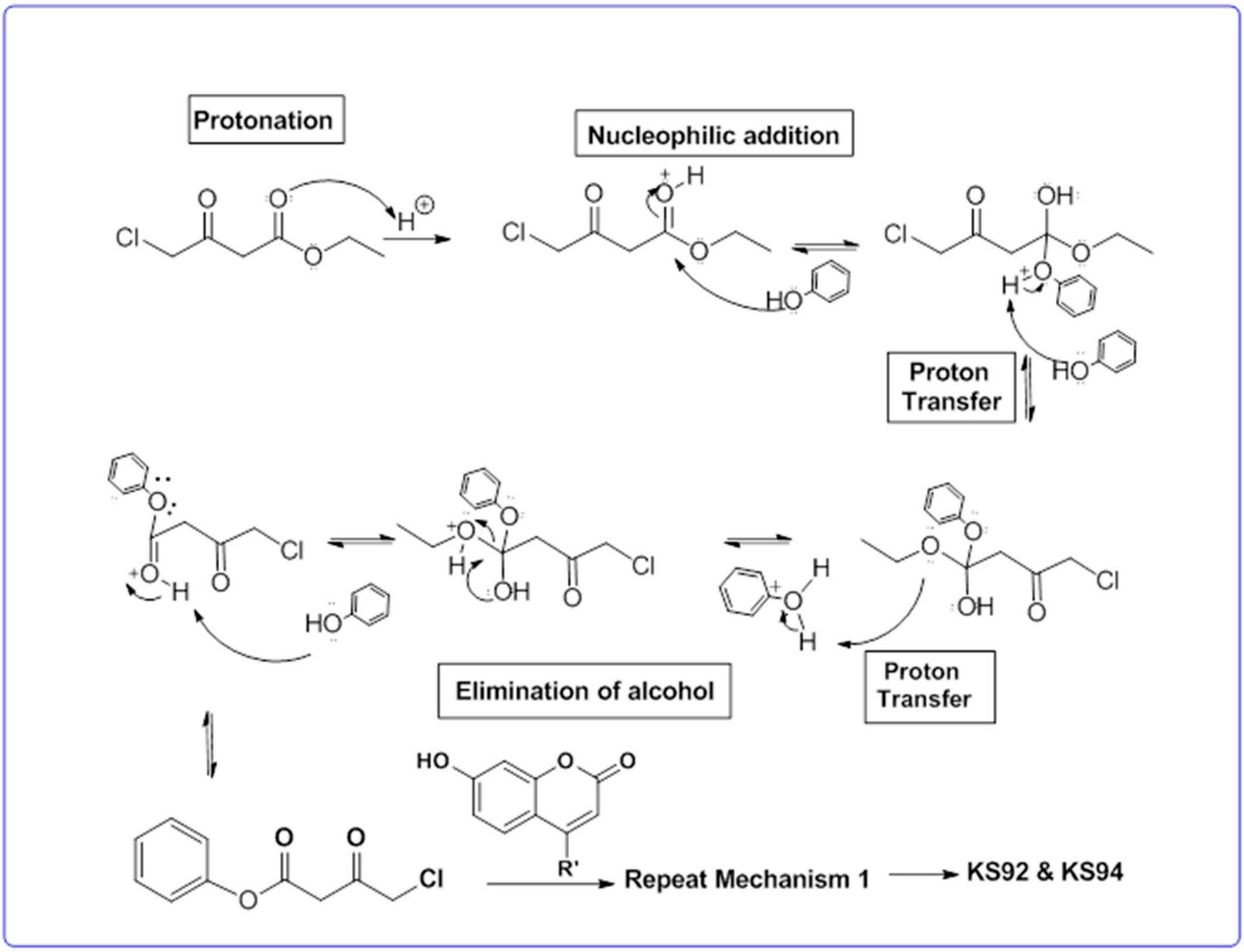

^{1}H-NMR Characterization of (KS-81) as representative compound
Ethyl 4-((4-methyl-2-oxo-2*H*-chromen-7-yl)oxy)-3-oxobutanoate (KS-81))

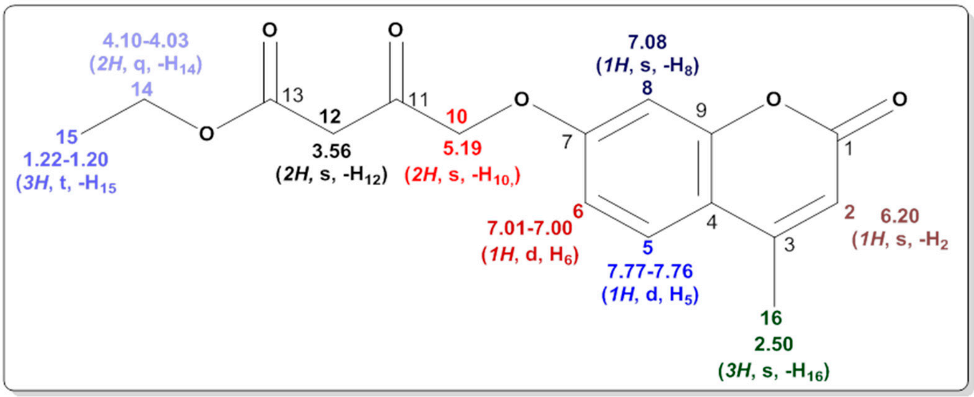

^{1}H-NMR (500 MHz, DMSO-d6, δ ppm): 7.77-7.76 (1H, d, *J*=3.9 Hz, -H5), 7.08 (1H, s, - -H8), 7.01-7.00 (1H, d, *J*=5.3 Hz, -H6), 6.20 (1H, s, -H2), 5.19 (2H, s, -H10), 4.10-4.03 (2H, q, -H14`), 3.56 (2H, s, -H10), 2.50 (3H, s, -H16), 1.22-1.20 (3H, t, -H15)

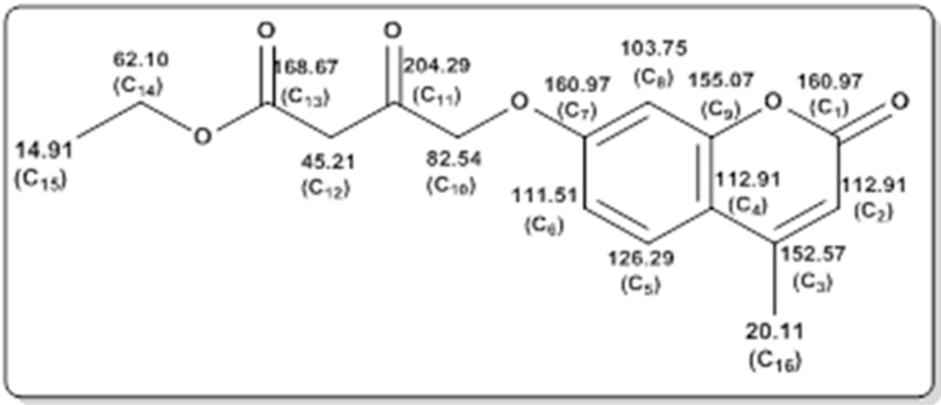
62.10
(C14)
168.67
(C13)
204.29
(C11)
103.75
(C8)
160.97
(C7)
155.07
(C9)
160.97
(C1)
14.91
(C15)
45.21
(C12)
82.54
(C10)
111.51
(C6)
112.91
(C4)
112.91
(C2)
126.29
(C5)
152.57
(C3)
20.11
(C16)

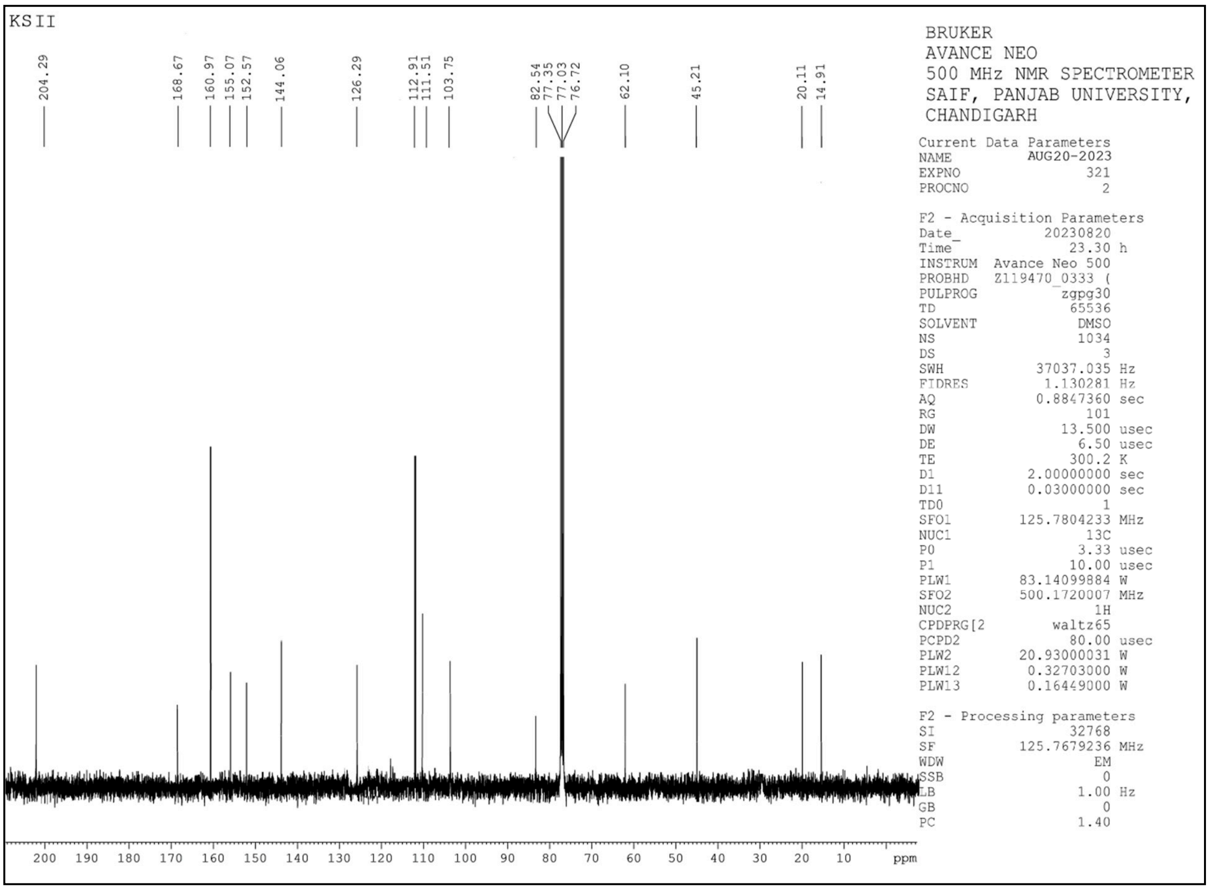
KSII
204.29
168.67
160.97
155.07
152.57
144.06
126.29
112.91
111.51
103.75
82.54
77.35
77.03
76.72
62.10
45.21
20.11
14.91
BRUKER
AVANCE NEO
500 MHz NMR SPECTROMETER
SAIF, PANJAB UNIVERSITY,
CHANDIGARH
Current Data Parameters
NAME AUG20-2023
EXPNO 321
PROCNO 2
F2 - Acquisition Parameters
Date_ 20230820
Time 23.30 h
INSTRUM Avance Neo 500
PROBHD Z119470_0333 (
PULPROG zgpg30
TD 65536
SOLVENT DMSO
NS 1034
DS 3
SWH 37037.035 Hz
FIDRES 1.130281 Hz
AQ 0.8847360 sec
RG 101
DW 13.500 usec
DE 6.50 usec
TE 300.2 K
D1 2.00000000 sec
D11 0.03000000 sec
TD0 1
SFO1 125.7804233 MHz
NUC1 13C
P0 3.33 usec
P1 10.00 usec
PLW1 83.14099884 W
SFO2 500.1720007 MHz
NUC2 1H
CPDPRG[2 waltz65
PCPD2 80.00 usec
PLW2 20.93000031 W
PLW12 0.32703000 W
PLW13 0.16449000 W
F2 - Processing parameters
SI 32768
SF 125.7679236 MHz
WDW EM
SSB 0
LB 1.00 Hz
GB 0
PC 1.40
200 190 180 170 160 150 140 130 120 110 100 90 80 70 60 50 40 30 20 10 ppm

KS-80

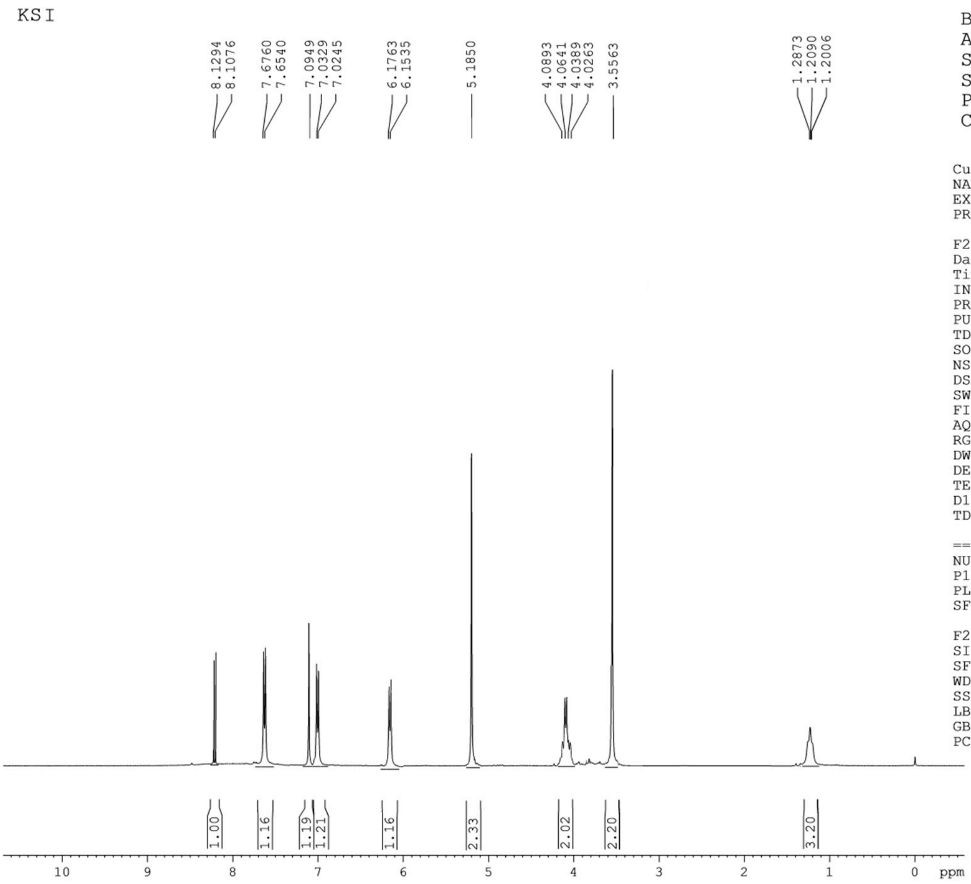

BRUKER
AVANCE II 500 NMR
Spectrometer
SAIF
Panjab University
Chandigarh

Current Data Parameters
NAME AUG20-2023
EXPNO 500
PROCNO 1

F2 - Acquisition Parameters
Date_ 20230820
Time 0.43
INSTRUM spect
PROBHD 5 mm PABBO BB-
PULPROG zg30
TD 65533
SOLVENT DMSO
NS 8
DS 3
SWH 12039.230 Hz
FIDRES 0.183399 Hz
AQ 2.7263477 sec
RG 436
DW 41.600 usec
DE 6.00 usec
TE 294.7 K
D1 1.00000000 sec
TD0 1

======== CHANNEL f1 ========
NUC1 1H
P1 10.90 usec
PL1 -3.00 dB
SFO1 400.1324710 MHz

F2 - Processing parameters
SI 32768
SF 500.1300080 MHz
WDW EM
SSB 0
LB 0.30 Hz
GB 0
PC 1.00

KSI

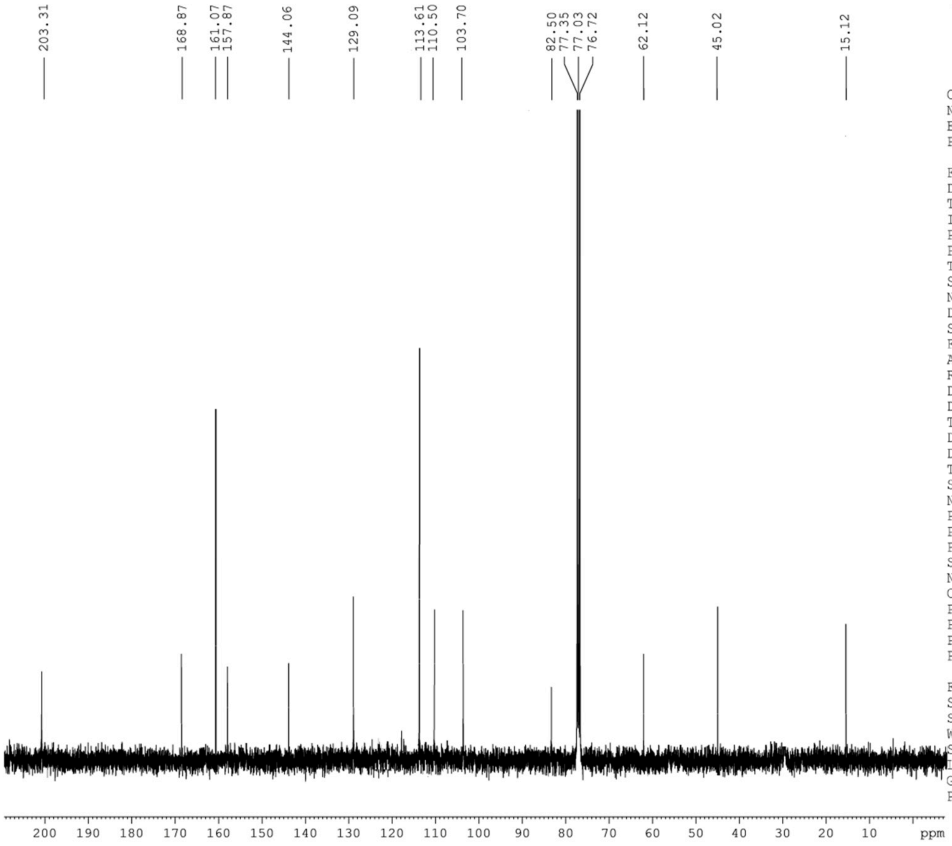

BRUKER
AVANCE NEO
500 MHz NMR SPECTROMETER
SAIF, PANJAB UNIVERSITY,
CHANDIGARH

Current Data Parameters

NAME	AUG20-2023	
EXPNO	301	
PROCNO	1	

F2 - Acquisition Parameters

Date_	20230820	
Time	22.04	h
INSTRUM	Avance Neo 500	
PROBHD	Z119470_0333 (	
PULPROG	zgpg30	
TD	65536	
SOLVENT	DMSO	
NS	1034	
DS	3	
SWH	37037.035	Hz
FIDRES	1.130281	Hz
AQ	0.8847360	sec
RG	101	
DW	13.500	usec
DE	6.50	usec
TE	300.2	K
D1	2.00000000	sec
D11	0.03000000	sec
TD0	1	
SFO1	125.7804233	MHz
NUC1	13C	
P0	3.33	usec
P1	10.00	usec
PLW1	83.14099884	W
SFO2	500.1720007	MHz
NUC2	1H	
CPDPRG[2	waltz65	
PCPD2	80.00	usec
PLW2	20.93000031	W
PLW12	0.32703000	W
PLW13	0.16449000	W

F2 - Processing parameters

SI	32768	
SF	125.7679236	MHz
WDW	EM	
SSB	0	
LB	1.00	Hz
GB	0	
PC	1.40	

SAIF,PANJAB UNIVERSITY,CHANDIGARH

SYNAPT-XS#DBA064

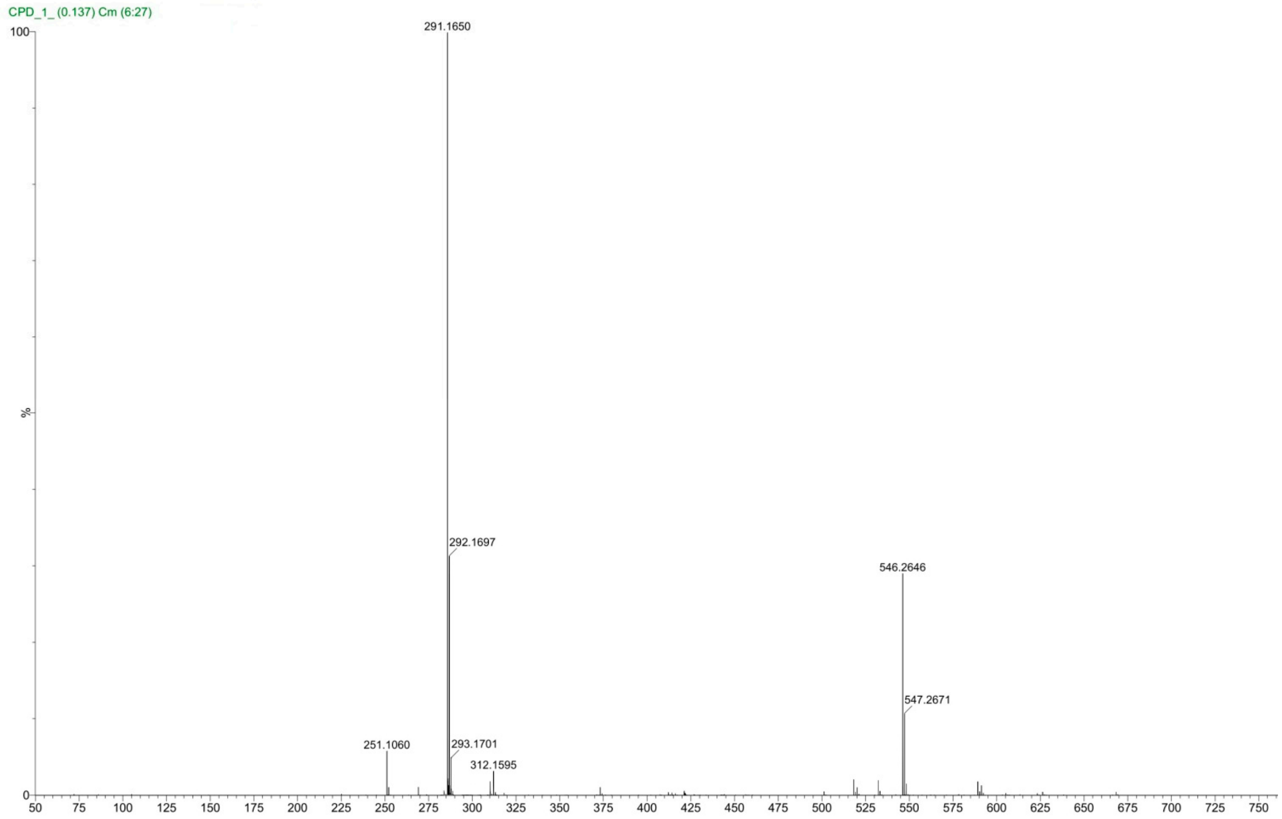

KS 81

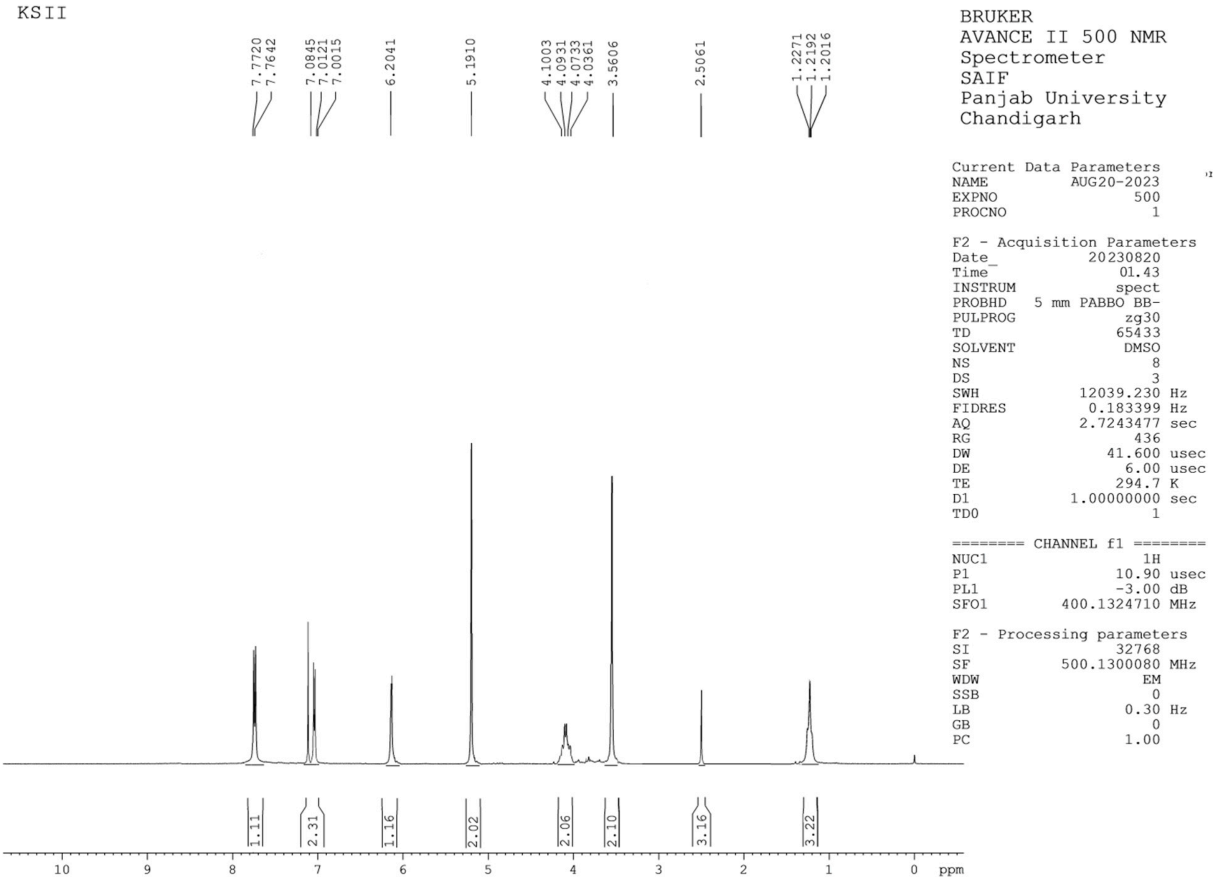
KSII
7.7720
7.7642
7.0845
7.0121
7.0015
6.2041
5.1910
4.1003
4.0931
4.0733
4.0361
3.5606
2.5061
1.2271
1.2192
1.2016
1.11
2.31
1.16
2.02
2.06
2.10
3.16
3.22
10 9 8 7 6 5 4 3 2 1 0 ppm
BRUKER
AVANCE II 500 NMR
Spectrometer
SAIF
Panjab University
Chandigarh
Current Data Parameters
NAME AUG20-2023
EXPNO 500
PROCNO 1
F2 - Acquisition Parameters
Date_ 20230820
Time 01.43
INSTRUM spect
PROBHD 5 mm PABBO BB-
PULPROG zg30
TD 65433
SOLVENT DMSO
NS 8
DS 3
SWH 12039.230 Hz
FIDRES 0.183399 Hz
AQ 2.7243477 sec
RG 436
DW 41.600 usec
DE 6.00 usec
TE 294.7 K
D1 1.00000000 sec
TD0 1
======== CHANNEL f1 ========
NUC1 1H
P1 10.90 usec
PL1 -3.00 dB
SFO1 400.1324710 MHz
F2 - Processing parameters
SI 32768
SF 500.1300080 MHz
WDW EM
SSB 0
LB 0.30 Hz
GB 0
PC 1.00

KSII

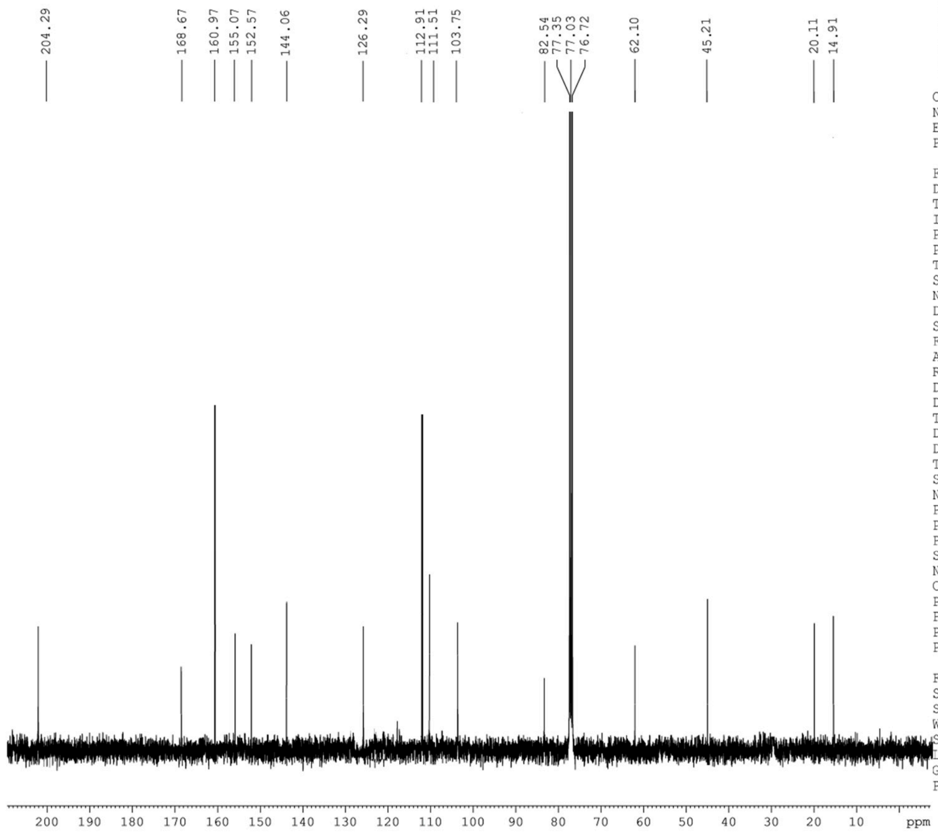

BRUKER
AVANCE NEO
500 MHz NMR SPECTROMETER
SAIF, PANJAB UNIVERSITY,
CHANDIGARH

Current Data Parameters

NAME	AUG20-2023
EXPNO	321
PROCNO	2

F2 - Acquisition Parameters

Date_	20230820
Time	23.30 h
INSTRUM	Avance Neo 500
PROBHD	Z119470_0333 (
PULPROG	zgpg30
TD	65536
SOLVENT	DMSO
NS	1034
DS	3
SWH	37037.035 Hz
FIDRES	1.130281 Hz
AQ	0.8847360 sec
RG	101
DW	13.500 usec
DE	6.50 usec
TE	300.2 K
D1	2.00000000 sec
D11	0.03000000 sec
TD0	1
SFO1	125.7804233 MHz
NUC1	13C
P0	3.33 usec
P1	10.00 usec
PLW1	83.14099884 W
SFO2	500.1720007 MHz
NUC2	1H
CPDPRG[2	waltz65
PCPD2	80.00 usec
PLW2	20.93000031 W
PLW12	0.32703000 W
PLW13	0.16449000 W

F2 - Processing parameters

SI	32768
SF	125.7679236 MHz
WDW	EM
SSB	0
LB	1.00 Hz
GB	0
PC	1.40

WATERS, Q-TOF MICROMASS (LC-MS)

SAIF/CIL,PANJAB UNIVERSITY,CHANDIGARH

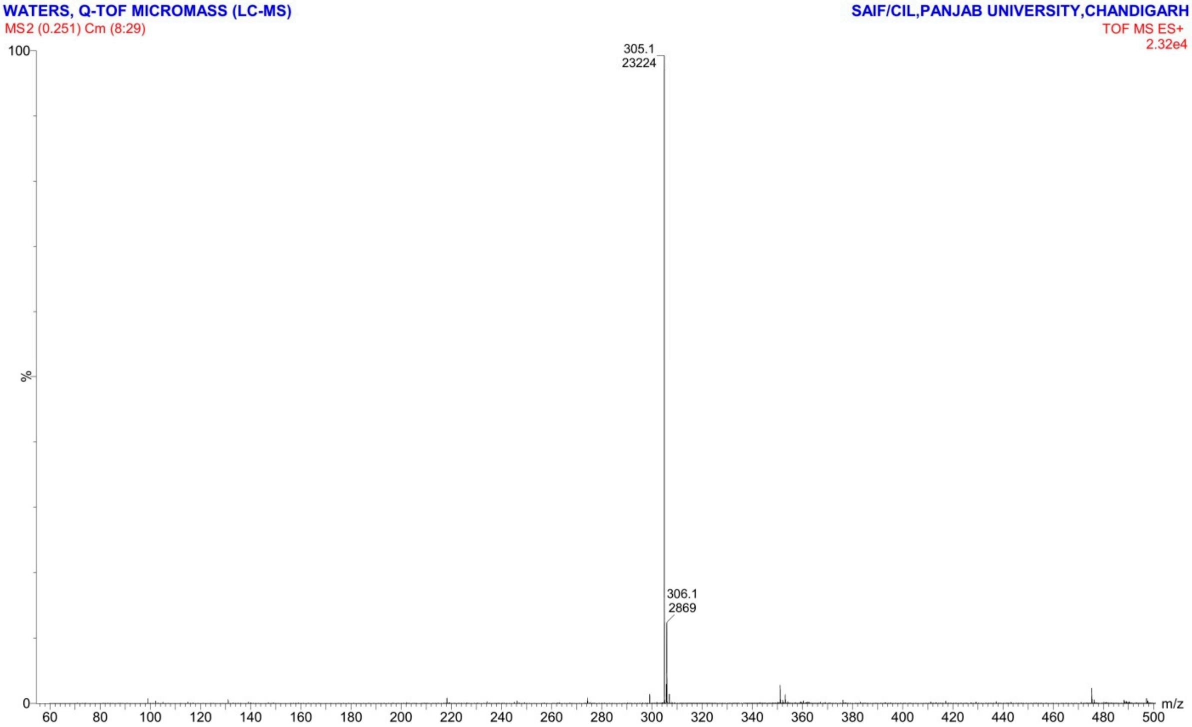

KS 82

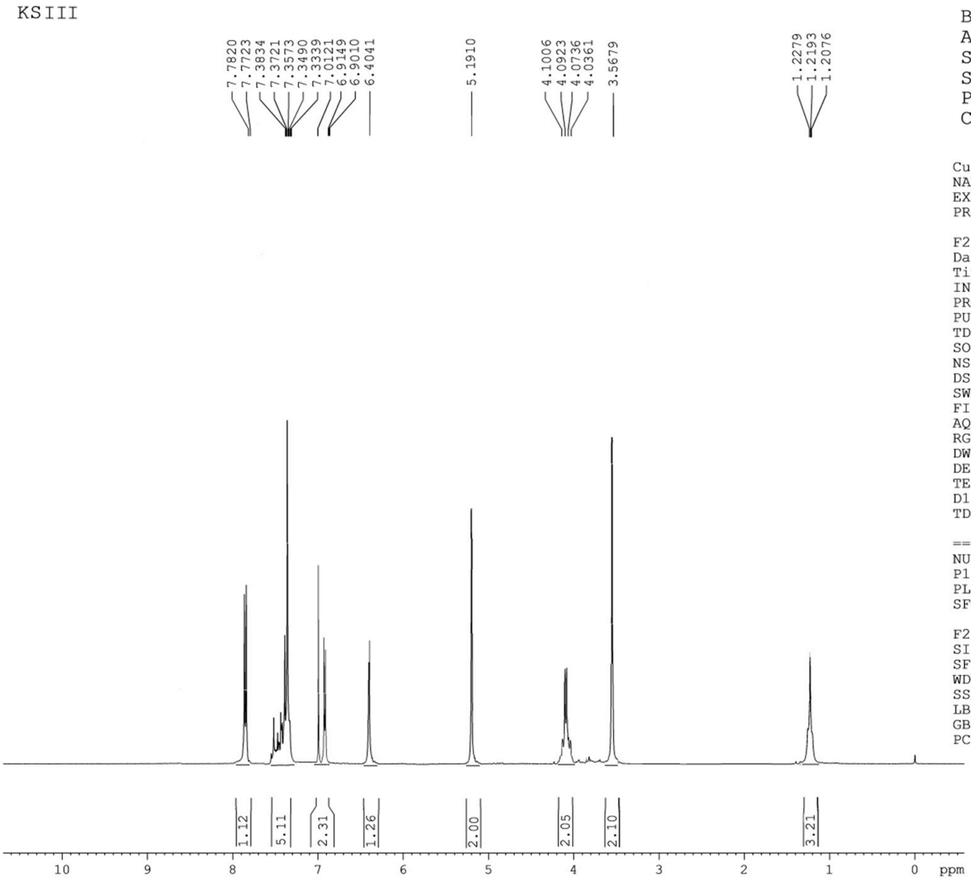

BRUKER
AVANCE II 500 NMR
Spectrometer
SAIF
Panjab University
Chandigarh

Current Data Parameters

NAME	AUG28-2023
EXPNO	500
PROCNO	1

F2 - Acquisition Parameters

Date_	20230828
Time	01.48
INSTRUM	spect
PROBHD	5 mm PABBO BB-
PULPROG	zg30
TD	65433
SOLVENT	DMSO
NS	9
DS	3
SWH	12099.237 Hz
FIDRES	0.183399 Hz
AQ	2.9243479 sec
RG	436
DW	41.600 usec
DE	6.00 usec
TE	294.7 K
D1	1.00000000 sec
TD0	1

======== CHANNEL f1 ========

NUC1	1H
P1	10.90 usec
PL1	-3.00 dB
SFO1	400.1324710 MHz

F2 - Processing parameters

SI	32767
SF	500.1700080 MHz
WDW	EM
SSB	0
LB	0.30 Hz
GB	0
PC	1.00

KSIII

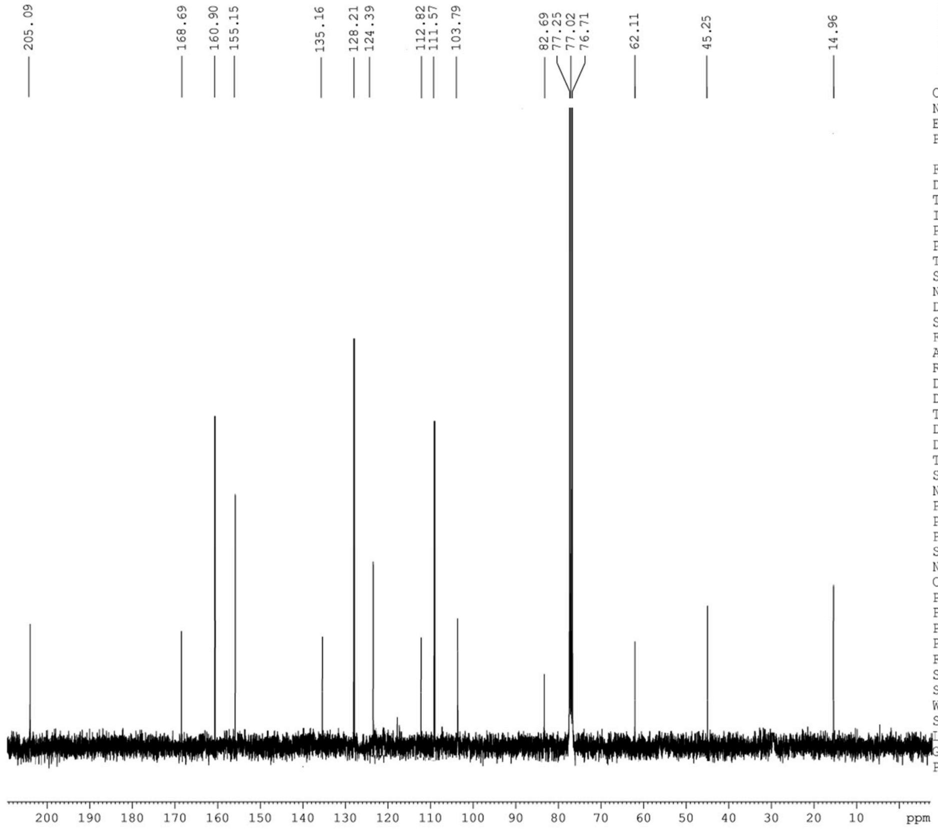

BRUKER
AVANCE NEO
500 MHz NMR SPECTROMETER
SAIF, PANJAB UNIVERSITY,
CHANDIGARH

Current Data Parameters

NAME	AUG28-2023
EXPNO	323
PROCNO	3

F2 - Acquisition Parameters

Date_	20230828
Time	23.80 h
INSTRUM	Avance Neo 500
PROBHD	Z119470_0383
PULPROG	zgpg30
TD	65533
SOLVENT	DMSO
NS	1035
DS	3
SWH	37037.035 Hz
FIDRES	1.130281 Hz
AQ	0.8847360 sec
RG	101
DW	13.500 usec
DE	6.50 usec
TE	300.2 K
D1	2.00000000 sec
D11	0.03000000 sec
TD0	1
SFO1	125.7804233 MHz
NUC1	13C
P0	3.33 usec
P1	10.00 usec
PLW1	83.14099884 W
SFO2	500.1720007 MHz
NUC2	1H
CPDPRG[2	waltz65
PCPD2	80.00 usec
PLW2	20.93000031 W
PLW12	0.32703000 W
PLW13	0.16449000 W

F2 - Processing parameters

SI	32768
SF	125.7679236 MHz
WDW	EM
SSB	0
LB	1.00 Hz
GB	0
PC	1.40

WATERS, Q-TOF MICROMASS (ESI-MS)

SAIF/CIL,PANJAB UNIVERSITY,CHANDIGARH

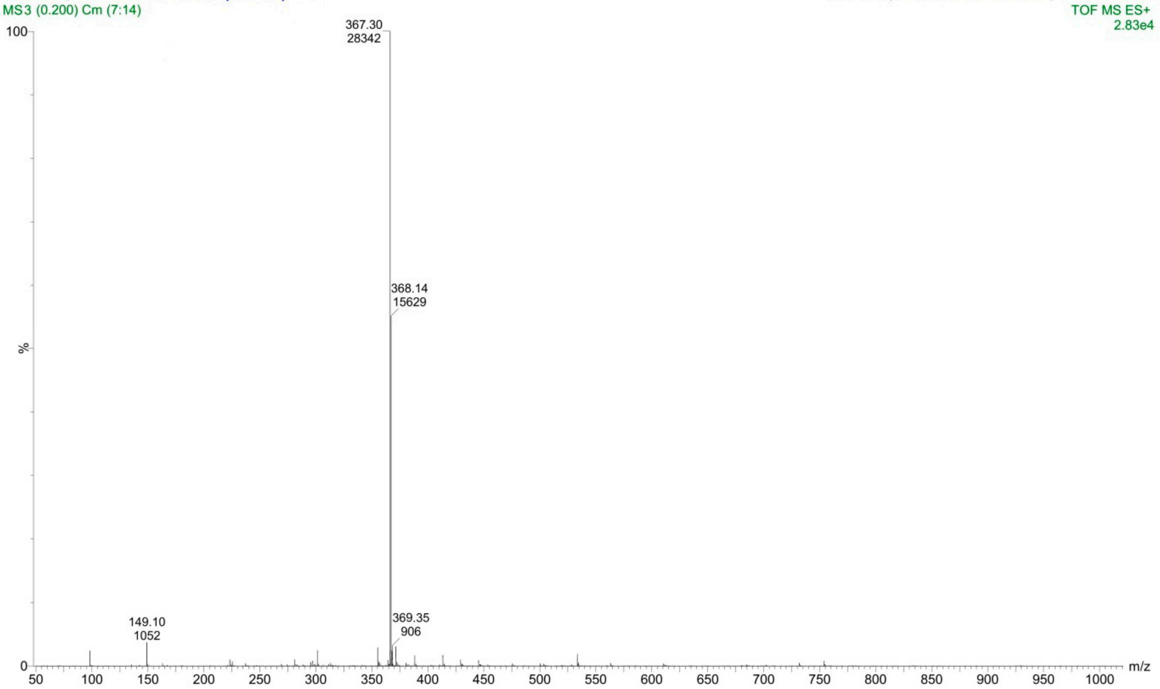

KS92

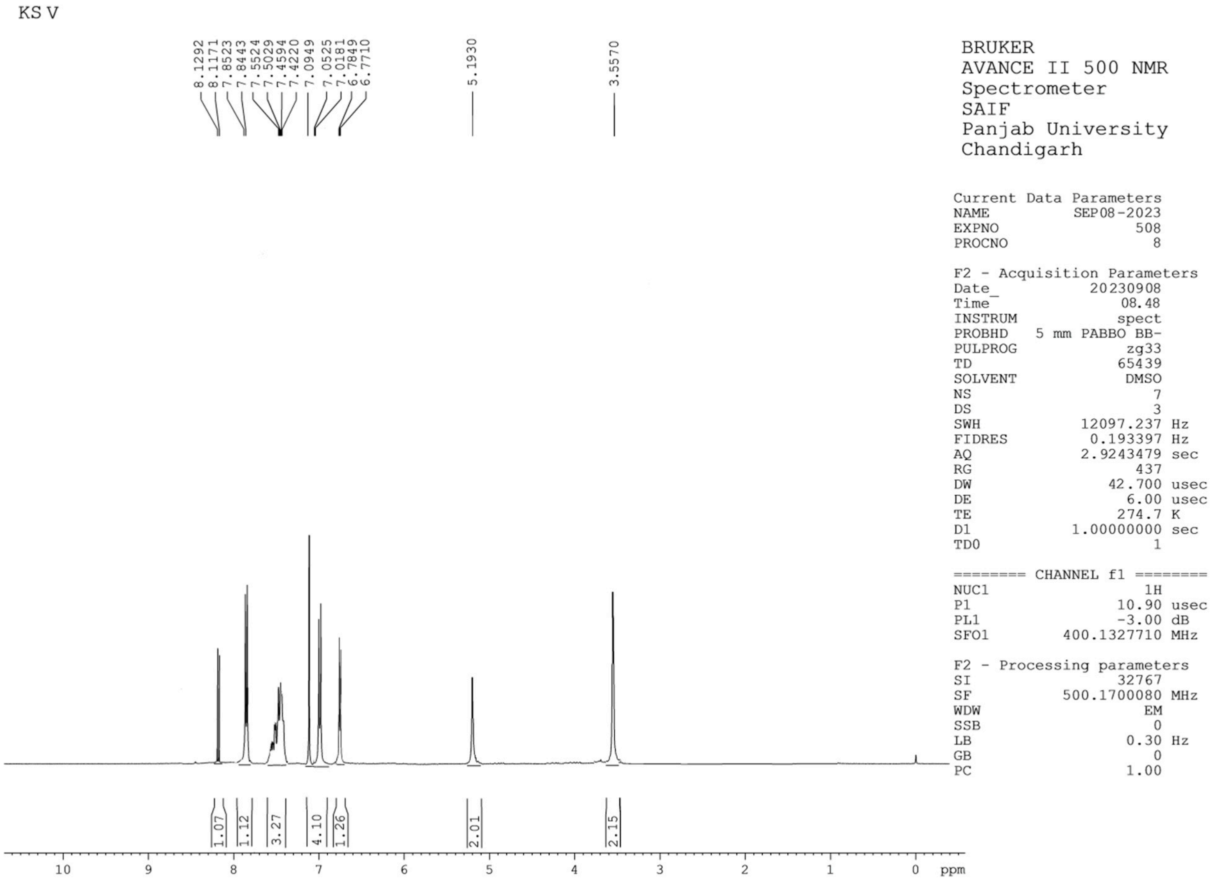

BRUKER
AVANCE II 500 NMR
Spectrometer
SAIF
Panjab University
Chandigarh

Current Data Parameters
NAME SEP08-2023
EXPNO 508
PROCNO 8

F2 - Acquisition Parameters
Date_ 20230908
Time 08.48
INSTRUM spect
PROBHD 5 mm PABBO BB-
PULPROG zg33
TD 65439
SOLVENT DMSO
NS 7
DS 3
SWH 12097.237 Hz
FIDRES 0.193397 Hz
AQ 2.9243479 sec
RG 437
DW 42.700 usec
DE 6.00 usec
TE 274.7 K
D1 1.00000000 sec
TD0 1

======== CHANNEL f1 ========
NUC1 1H
P1 10.90 usec
PL1 -3.00 dB
SFO1 400.1327710 MHz

F2 - Processing parameters
SI 32767
SF 500.1700080 MHz
WDW EM
SSB 0
LB 0.30 Hz
GB 0
PC 1.00

KSIV

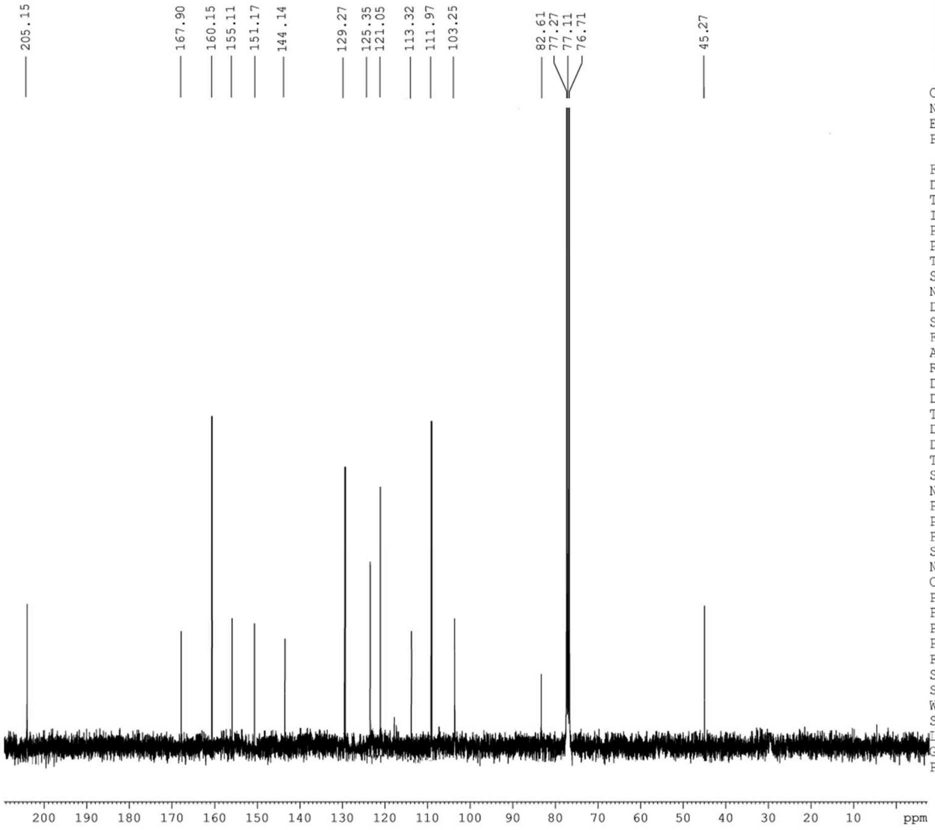

BRUKER
AVANCE NEO
500 MHz NMR SPECTROMETER
SAIF, PANJAB UNIVERSITY,
CHANDIGARH

Current Data Parameters
NAME SEP05-2023
EXPNO 323
PROCNO 3

F2 - Acquisition Parameters
Date_ 20230905
Time 22.80 h
INSTRUM Avance Neo 500
PROBHD Z119470_0383
PULPROG zgpg30
TD 65532
SOLVENT DMSO
NS 1025
DS 3
SWH 37037.035 Hz
FIDRES 1.130281 Hz
AQ 0.8847360 sec
RG 101
DW 13.500 usec
DE 6.50 usec
TE 300.2 K
D1 2.00000000 sec
D11 0.03000000 sec
TD0 1
SFO1 125.7804233 MHz
NUC1 13C
P0 3.33 usec
P1 10.00 usec
PLW1 83.14099884 W
SFO2 500.1720007 MHz
NUC2 1H
CPDPRG[2 waltz65
PCPD2 80.00 usec
PLW2 20.93000031 W
PLW12 0.32703000 W
PLW13 0.16449000 W
F2 - Processing parameters
SI 32768
SF 125.7679236 MHz
WDW EM
SSB 0
LB 1.00 Hz
GB 0
PC 1.40

WATERS, Q-TOF MICROMASS (ESI-MS) **SAIF/CIL,PANJAB UNIVERSITY,CHANDIGARH**

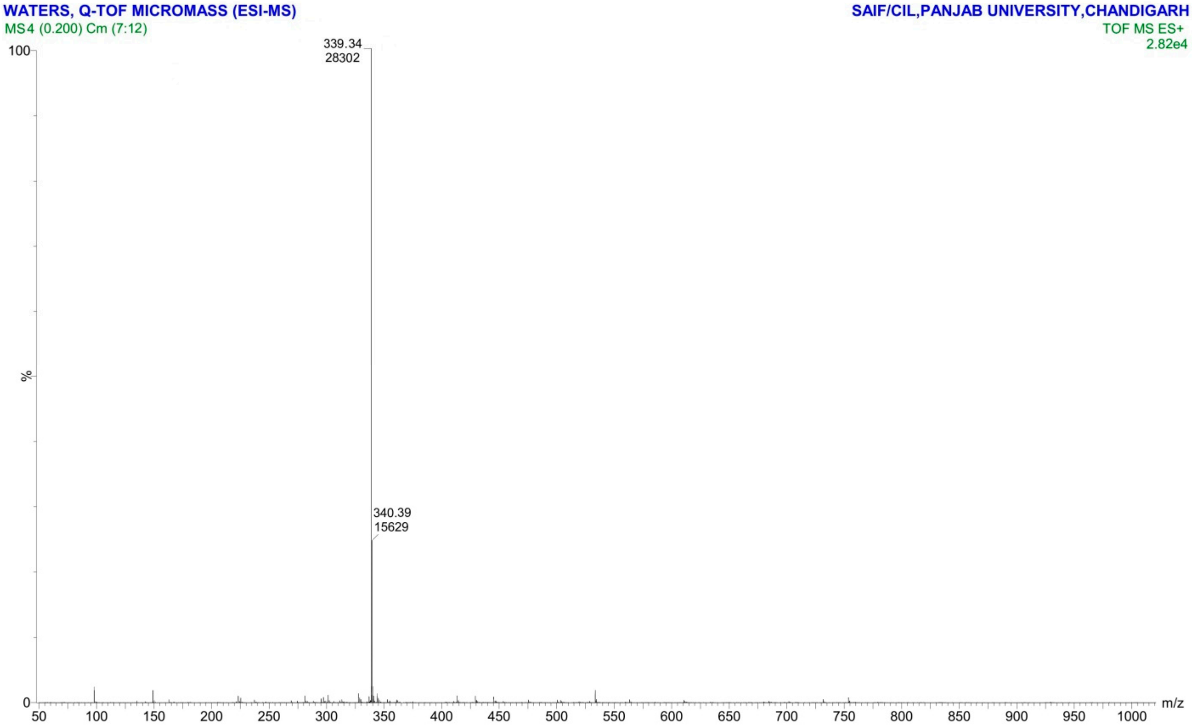

KS94

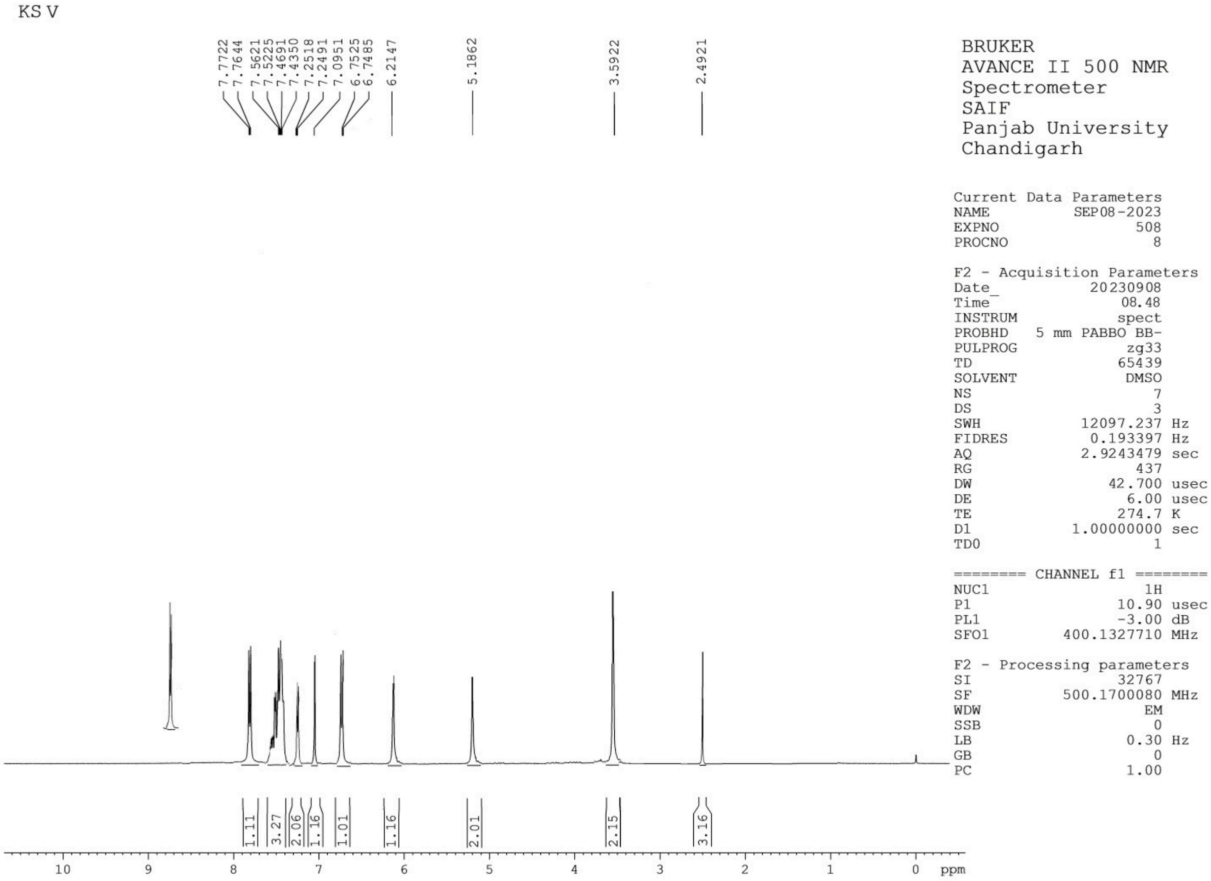

BRUKER
AVANCE II 500 NMR
Spectrometer
SAIF
Panjab University
Chandigarh

Current Data Parameters
NAME SEP08-2023
EXPNO 508
PROCNO 8

F2 - Acquisition Parameters
Date_ 20230908
Time 08.48
INSTRUM spect
PROBHD 5 mm PABBO BB-
PULPROG zg33
TD 65439
SOLVENT DMSO
NS 7
DS 3
SWH 12097.237 Hz
FIDRES 0.193397 Hz
AQ 2.9243479 sec
RG 437
DW 42.700 usec
DE 6.00 usec
TE 274.7 K
D1 1.00000000 sec
TD0 1

======== CHANNEL f1 ========
NUC1 1H
P1 10.90 usec
PL1 -3.00 dB
SFO1 400.1327710 MHz

F2 - Processing parameters
SI 32767
SF 500.1700080 MHz
WDW EM
SSB 0
LB 0.30 Hz
GB 0
PC 1.00

KS V

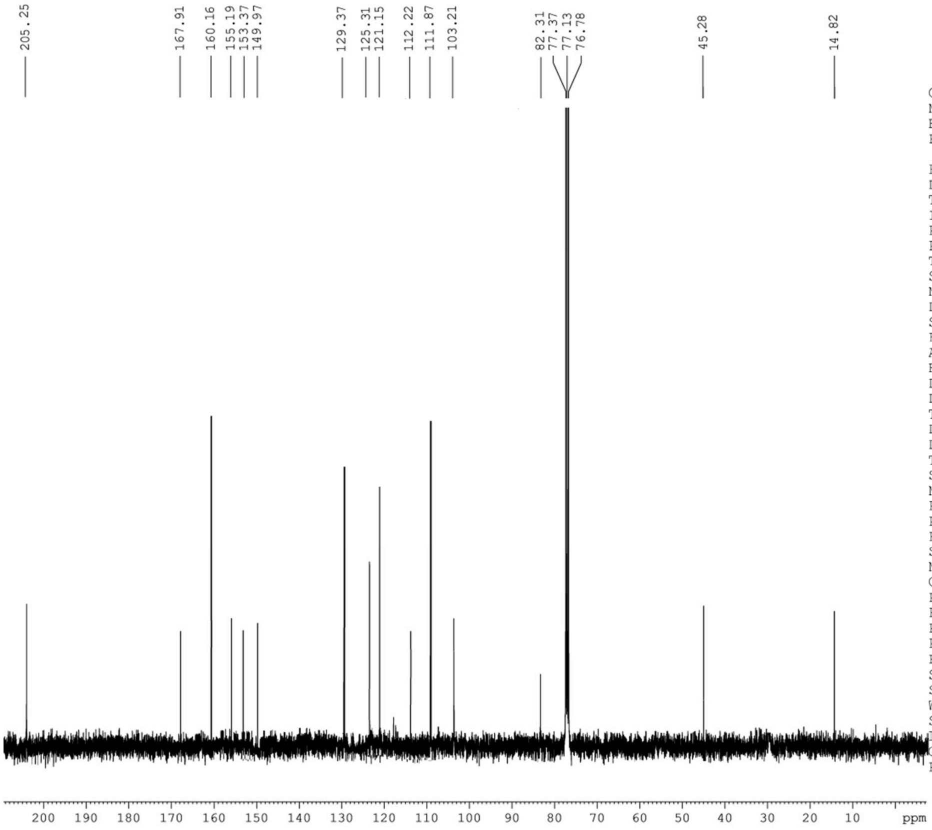

BRUKER
AVANCE NEO
500 MHz NMR SPECTROMETER
SAIF, PANJAB UNIVERSITY,
CHANDIGARH

Current Data Parameters

NAME	SEP05-2023
EXPNO	325
PROCNO	5

F2 - Acquisition Parameters

Date_	20230905
Time	22.50 h
INSTRUM	Avance Neo 500
PROBHD	Z119470_0385
PULPROG	zgpg30
TD	65532
SOLVENT	DMSO
NS	1055
DS	3
SWH	37037.535 Hz
FIDRES	1.130281 Hz
AQ	0.8847360 sec
RG	101
DW	15.500 usec
DE	6.50 usec
TE	300.2 K
D1	2.00000000 sec
D11	0.03000000 sec
TD0	1
SFO1	125.7805233 MHz
NUC1	13C
P0	3.33 usec
P1	10.00 usec
PLW1	83.15099884 W
SFO2	500.1720007 MHz
NUC2	1H
CPDPRG[2	waltz65
PCPD2	80.00 usec
PLW2	20.93000031 W
PLW12	0.32703000 W
PLW13	0.16459000 W

F2 - Processing parameters

SI	32768
SF	125.7679236 MHz
WDW	EM
SSB	0
LB	1.00 Hz
GB	0
PC	1.40

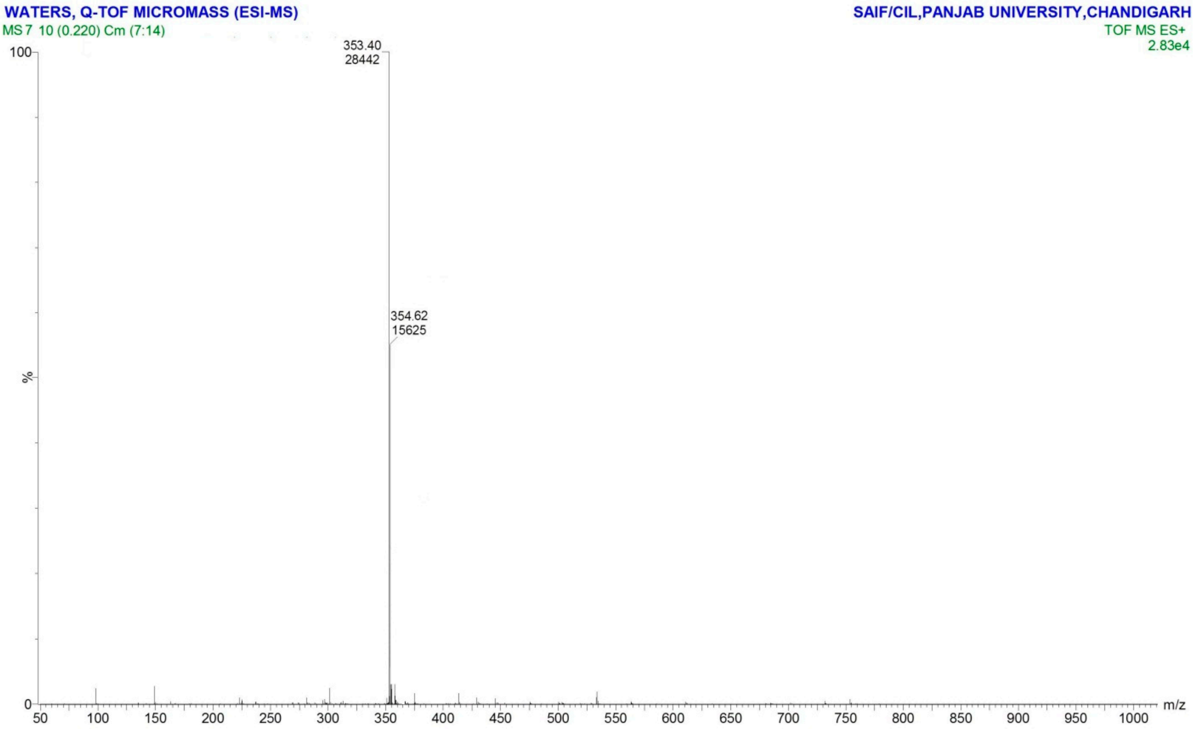
WATERS, Q-TOF MICROMASS (ESI-MS)
MS 7 10 (0.220) Cm (7:14)
SAIF/CIL,PANJAB UNIVERSITY,CHANDIGARH
TOF MS ES+
2.83e4
353.40
28442
354.62
15625
100
%
0
50 100 150 200 250 300 350 400 450 500 550 600 650 700 750 800 850 900 950 1000
m/z
